# Formation of electron traps in semiconducting polymers via a slow triple-encounter between trap precursor particles

**DOI:** 10.1080/14686996.2024.2312148

**Published:** 2024-01-31

**Authors:** Mohammad Sedghi, Camilla Vael, Wei-Hsu Hu, Michael Bauer, Daniele Padula, Alessandro Landi, Mirko Lukovic, Matthias Diethelm, Gert-Jan Wetzelaer, Paul W. M. Blom, Frank Nüesch, Roland Hany

**Affiliations:** a Laboratory for Functional Polymers, Empa, Swiss Federal Laboratories for Materials Science and Technology, Dübendorf, Switzerland; b Institute of Materials Science and Engineering, Ecole Polytechnique Fédérale de Lausanne (EPFL), Lausanne, Switzerland; c Fluxim AG, Winterthur, Switzerland; d Dipartimento di Biotecnologie, Chimia e Farmacia, Università di Siena, Siena, Italy; e Dipartimento di Chimica e Biologia “Adolfo Zambelli”, University of Salerno, Salerno, Italy; f Cellulose & Wood Materials, Empa, Swiss Federal Laboratories for Materials Science and Technology, Dübendorf, Switzerland; g Max Planck Institute for Polymer Research, Mainz, Germany

**Keywords:** Polymer light-emitting diodes, electron trap, charge trap dynamics, device physics, semiconducting polymer

## Abstract

Already in 2012, Blom et al. reported (Nature Materials 2012, *11*, 882) in semiconducting polymers on a general electron-trap density of ≈3 × 10^17^ cm^−3^, centered at an energy of ≈3.6 eV below vacuum. It was suggested that traps have an extrinsic origin, with the water-oxygen complex [2(H_2_O)-O_2_] as a possible candidate, based on its electron affinity. However, further evidence is lacking and the origin of universal electron traps remained elusive. Here, in polymer diodes, the temperature-dependence of reversible electron traps is investigated that develop under bias stress slowly over minutes to a density of 2 × 10^17^ cm^−3^, centered at an energy of 3.6 eV below vacuum. The trap build-up dynamics follows a 3^rd^-order kinetics, in line with that traps form via an encounter between three diffusing precursor particles. The accordance between universal and slowly evolving traps suggests that general electron traps in semiconducting polymers form via a triple-encounter process between oxygen and water molecules that form the suggested [2(H_2_O)-O_2_] complex as the trap origin.

## IMPACT STATEMENT

Formation of universal electron traps in polymer light-emitting diodes is a dynamic process that occurs via a slow triple-encounter between trap precursor species, with the water-oxygen [2(H_2_O)-O_2_] complex as a likely candidate.

## GRAPHICAL ABSTRACT

**Figure:**
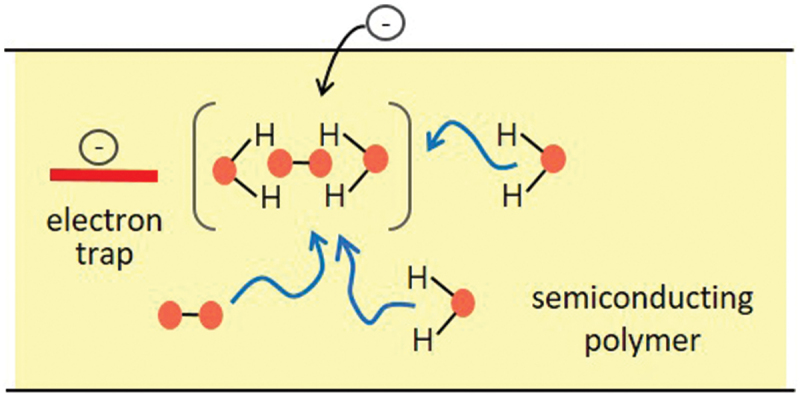

## 1. Introduction

Semiconducting polymers are attractive candidates for realizing solution-processed organic solar cells [1], field-effect transistors [2] or polymer light-emitting diodes (PLEDs) [3]. Crucial for efficient device operation are the charge-transport properties of electrons and holes. For PLEDs, high device performance requires a balanced charge transport between electrons and holes. For most semiconducting polymers in pristine devices [4], however, the magnitude of the electron current is found to be considerably lower than the hole current, which is generally attributed to the presence of electron traps situated within the bandgap [5–9].

Electron traps have a negative effect on the device performance in several ways. First, non-radiative recombination between trapped electrons and free holes competes with the emissive bimolecular Langevin recombination. In addition, the photoluminescence quantum yield is lowered due to excitons that diffuse towards traps and are quenched after their formation. Furthermore, electron trapping confines the charge recombination in a region close to the cathode. This results in quenching of excitons by the metallic electrode. In general, this confinement also reduces the light outcoupling efficiency, because in a PLED the emitting layer is sandwiched between a weakly reflecting substrate electrode and a strongly reflecting metallic electrode, resulting in pronounced optical interference effects as function of the emitter position and the active film thickness [3,10].

Already more than 10 years ago, it has been reported that a universal electron-trap distribution exists in a wide range of polymers [5–7]. This suggests that the traps share a common extrinsic origin and are not due to intrinsic material-specific defects, such as synthetic impurities or twists and kinks in the polymer backbone. As a common origin for these omnipresent electron traps, oxygen, water and hydrated oxygen complexes have been identified, with [2(H_2_O)-O_2_] as a likely candidate, based on its electron affinity [5,6,11–13]. Implicitly, it has been assumed that universal electron traps are permanently present in the materials. Trap filling is an energetic downhill process [5] and therefore is expected to be fast. This then implies that also trapping by the general electron traps in semiconducting polymers should be fast, with a typical timescale for trap filling of ≈200 µs [14].

In contrast to this general notion, recently, electron traps have been identified in a number of polymers that develop slowly under bias stress, and trap formation proceeds over many minutes [14]. Such a result is not consistent with trap filling of permanent traps, but rather indicates that traps continuously form over time. It has been suggested that trap formation proceeds via an encounter complex created by slowly diffusing particles, which after formation is rapidly trapped by an electron. Here, we study the temperature-dependence of the slow electron-trap dynamics using electron-only devices and PLEDs under bias stress. We evaluate the trap density over time from the decaying current via numerical drift-diffusion simulation and find that the dynamics of trap formation clearly follows a 3^rd^-order kinetics. We argue that the suggested universal trap precursor [2(H_2_O)-O_2_] is consistent with the idea that three precursor particles form an encounter complex during diffusion. We conclude that general electron traps in semiconducting polymers are not permanently present but form slowly over time.

The polymer used as a reference in this work is a phenyl-substituted poly(*para*-phenylene vinylene) (PPV) copolymer termed super yellow (SY) [15,16]. We use SY as a model material because all relevant charge transport parameters have been experimentally determined [17,18]. We note that the basic features of slow and reversible electron trap formation have been demonstrated for other semiconducting polymers before, e.g. for poly(2-methoxy-5-(2-ethylhexyloxy)-1,4-phenylene vinylene (MEH-PPV) and poly(3-hexylthiophene) (P3HT) [14]. MEH-PPV is structurally related to the amorphous SY polymer, but P3HT is partially crystalline and belongs to a different polymer family. It thus appears that our results presumably are not restricted to SY but apply to a broad range of polymers.

## 2. Materials and methods

To fabricate PLEDs, indium tin oxide (ITO) coated glass substrates (≈11 Ohms square^−1^) were cleaned successively in acetone, ethanol, a 2 vol-% aqueous solution of Hellmanex and deionized water. 40-nm-thick poly(3,4-ethylenedioxythiophene) polystyrene sulfonate (PEDOT:PSS, Al 4083 from Ossila) films were spin-coated (1000 rpm s^−1^, 60 s at 3000 rpm) from filtered (pore size 0.45 μm) solutions and were dried for 10 min at 120°C. Then, the glass/ITO/PEDOT:PSS substrates were transferred in a glovebox (O_2_ < 5 ppm, H_2_O < 1 ppm) and were again heated for 10 min at 120°C. Dried (24 h, 0.1 mbar, 40°C) SY (Merck) was dissolved in a concentration of 5 mg mL^−1^ in anhydrous (H_2_O < 0.001%) toluene (Sigma-Aldrich). Solutions were stirred at least 12 h at 60°C inside the glovebox before coating. SY films with a thickness of (80 ± 5) nm were coated inside the glovebox from unfiltered solutions (2000 rpm s^−1^, 60 s at 2000 rpm) and were then dried at 60°C for 1 h. The cathode composed of calcium (Ca, 10 nm) and aluminum (Al, 70 nm) was subsequently thermally evaporated on top of the polymer through a shadow mask, defining eight cells per substrate with an active area of 3.1 or 7.1 mm^2^, respectively. For electron-only devices, PEDOT:PSS was replaced by a 20-nm-thick aluminum layer.

Devices were encapsulated using encapsulant coverslips and epoxy (E132 Ossila) that was cross-linked under UV illumination for 5 min. Temperature-dependent current characteristics were measured with encapsulated devices outside the glovebox using a Keithley 2400. Alternatively, non-encapsulated devices were measured directly inside the glovebox on a Paios measurement system (Fluxim AG, Switzerland, at 295 K). The temperature was controlled with a Peltier cooling/heating stage (ZTNG-100-B, Dr. Neumann, Peltier-Technik GmbH, Germany). To prevent the condensation of humidity and the formation of ice on the stage below 273 K, the setup was placed in a protective cover that was purged with a flow of N_2_. When measuring PLED transients, a preconditioning step at room temperature was applied that consisted of a current stress at 3.7 V for 7 min, followed by a relaxation time of 12 min. Electrical simulations were performed with Setfos 5.2 (Fluxim AG, Switzerland). For light-induced electron detrapping, we used an LED with a peak wavelength at 855 nm and a flux of 3.4 × 10^21^ m^−2^ s^−1^. Electrical simulation procedures and parameters are described in the Note 1, Supporting Information. Details of the kinetic Monte Carlo simulations are described in the Note 4, Supporting Information.

## 3. Results

**Figure 1.**
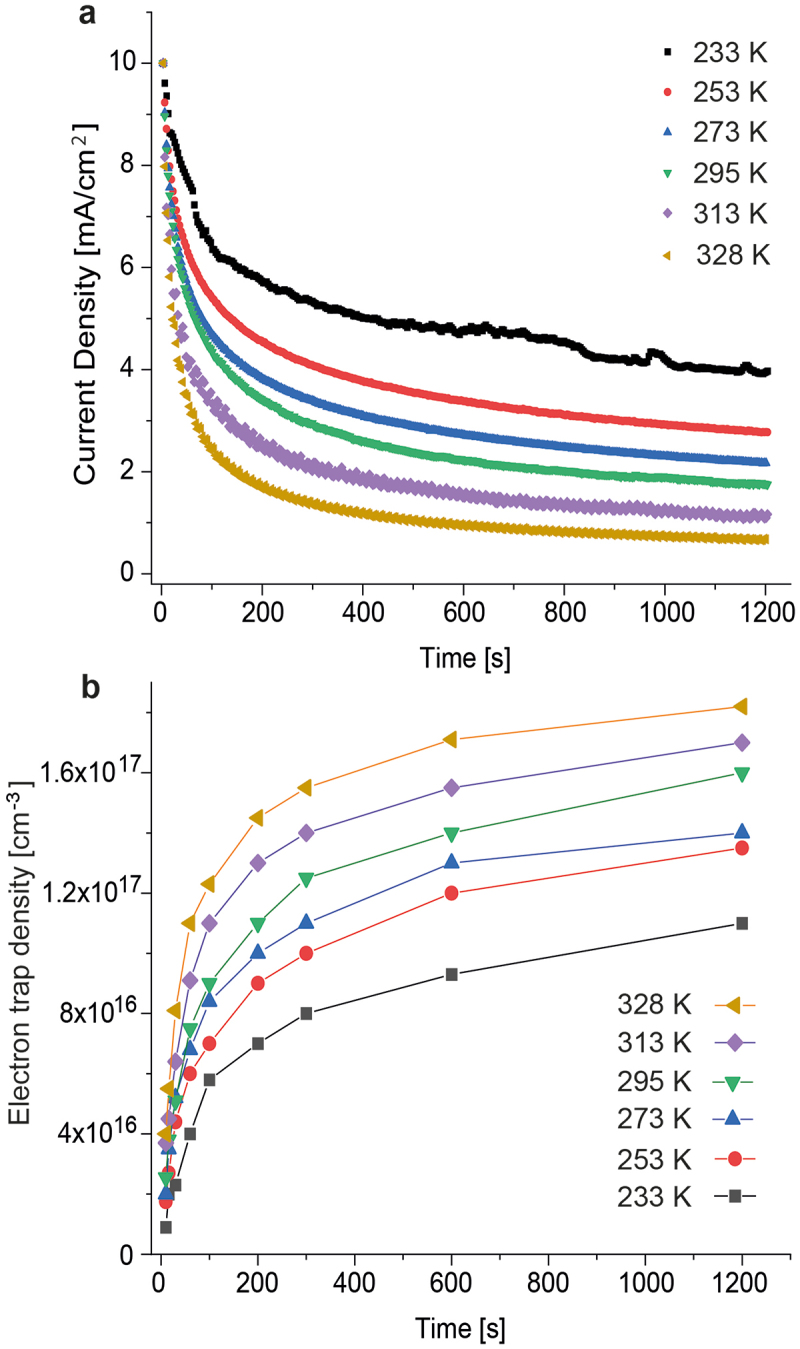
Electron current and trap formation at different temperatures. (a) Current decline for electron-only devices at different temperatures. The constant operating voltage varied between 3.7 V at 328 K and 5.5 V at 233 K. (b) Electron trap evolution at different temperatures. Each symbol is the result of a simulation, in which electron traps were added to match the measured current density.

Figure 1(a) displays the currents for ITO/Al/SY/Ca/Al electron-only devices for different temperatures. The voltage was first adjusted on the pristine device such that the current density was 10 mA cm^−2^, then the current was continuously measured during constant voltage operation. Currents are observed to decrease during operation, which we ascribe to the formation of electron traps. The initial current decay is strong for every temperature and levels off after around 200 s. At 328 K, the current reaches a plateau at ≈0.7 mA cm^−2^ after 1200 s of operation, which means that trap formation has stopped. With decreasing temperature, trap evolution slows down. By extrapolating the current decline for other temperatures down to the constant current level at 328 K, we estimate the time when no more traps form at other temperatures, such as after ≈4200 s at 295 K. We further show in Figure S1 of the Supporting Information that for every temperature the dynamics of the current decay, and thus the dynamics of trap formation is independent of the applied current density.

Currents were simulated using a drift-diffusion model with the extended Gaussian disorder model (EGDM) to describe the charge-carrier mobility [5,17,18]. First, for each temperature the experimental voltage and calculated charge mobility were slightly adjusted in the simulation to obtain the current density at switch-on. Afterward, the simulation parameters were kept constant, and trap sites were added gradually to match the current decay over time. The current decay is due to immobile trapped electron charge that displaces the flow of mobile electron charge. We simulated the trapped electron density *n(t)* assuming a trap-depth energy *E*_*t*_ of 0.65 eV, which corresponds to the trap depth of the universal electron traps in PPV polymers, *E*_*t*_ = 0.6–0.7 eV [5–7]. The simulation procedure is detailed in the Supporting Information Note 1.

In electron-only devices, the simulated current density depends very sensitively on the number of electron traps, and already for an added trap site density of 5 × 10^17^ cm^−3^, the current drops to <0.1 mA cm^−2^. Furthermore, the evolution of the simulated electron traps strongly depends on the temperature (Figure 1(b)). A trap density of ≈4 × 10^16^ cm^−3^ develops for every temperature during the first 60 s, but afterward the trap evolution slows down considerably with decreasing temperature. For example, the trap density is 1.2 × 10^17^ cm^−3^ after 100 s at 328 K, but this trap density has not developed even after 1200 s at 233 K. Because electron traps at 328 K have largely developed after 1200 s (Figure 1(a)), we estimate that the maximum electron trap density in the material that forms over time is ≈2 × 10^17^ cm^−3^. Trap filling of permanent traps is fast and depends little on the temperature (Note 1, Supporting Information). Therefore, the slowdown of the current decay at lower temperatures is not due to the slower filling of permanent traps by electrons, but indicates that trap formation slows down.

The question arises to what extent electron trap formation is a reversible process. If traps form via reduction of a weakly bound encounter complex between diffusing precursor species and the device is switched back to rest after drive, the particles separate again after electron detrapping. In this case, the same (slow) trap dynamics is observed when the device is switched on again. On the other hand, if the trap is stable after detrapping, traps fill immediately at switch on and the starting current of the subsequent measurement corresponds to the final current of the first measurement.

**Figure 2.**
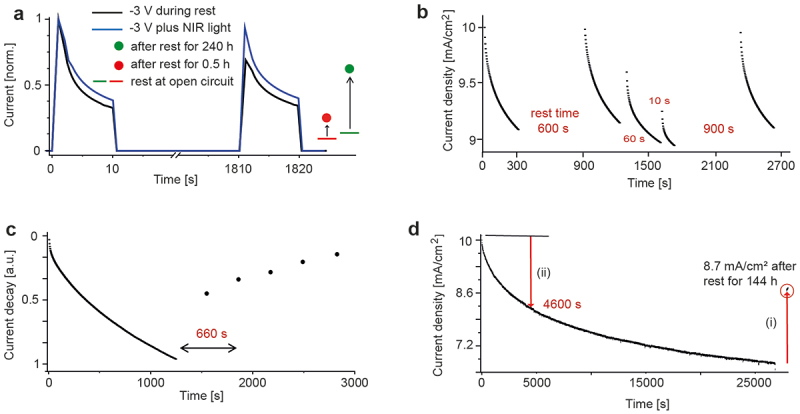
Dynamics of electron detrapping at 295 K. Current recovery in (a–d) during rest reflects the escape of trapped electrons via thermal emission, followed by trap disintegration via diffusive separation of trap precursor particles. (a) Current recovery of electron-only devices during rest at open circuit, when a reverse bias is applied, and when in addition NIR light is illuminated to release trapped electrons via photo-excitation. (b) Current evolution of a constant voltage-driven (3.7 V) PLED. The voltage stress was interrupted at particular moments and the device rested at 0 V for a certain time before switching to bias again. (c) Current recovery of a PLED after an operation time of 1200 s. Indicated is the detrapping time constant (*t* = 660 s), after which *n(t)* = 0.37 × *n(0)*. (d) Current decay of a PLED during an operation time of 7.5 h. The current recovery during subsequent storage (i) is due to disintegration of electron traps that have formed during 4600 s after switch-on (ii).

We operated both electron-only devices and PLEDs and examined the electron trap decay from the current recovery during subsequent rest periods (Figure 2). In electron-only devices (Figure 2(a)), detrapping occurs via thermal emission of electrons back to the conduction band of SY. For devices that were operated for 1200 s and were then stored at open circuit, current recovery was very slow (red, green symbols). Current recovery was faster when during rest (1800 s) between two voltage pulses (10 s, 2.5 V) an electric field was applied that helps to extract detrapped electrons to the external circuitry (black current trend). Finally, full current recovery was measured when in addition to the applied electric field during rest the device was illuminated with near-infrared (NIR, 855 nm) light (blue current trend). NIR light with a wavelength below the bandgap of SY photo-excites and thereby helps to release trapped electrons [14].

In PLEDs, the presence of free holes in connection with trapped electrons both during operation and after switch off must be clarified (for details see Note 2, Supporting Information). During operation, holes recombine with a fraction of trapped electrons (Shockley-Read-Hall, SRH, recombination). Therefore, the number of trapped electrons in a PLED is smaller than in an electron-only device. In addition, even for a large electron trap density, a substantial PLED current remains. In this case, the current is entirely due to the SRH recombination current between holes and trapped electrons. Therefore, for the same electron trap density, the current decay in a PLED is much smaller than in an electron-only device.

When a PLED is switched off, the majority of holes and free electrons recombine within a few microseconds (and the electroluminescence turns off [14]). Remaining free charges are extracted at the electrodes, but recombination between holes and trapped electrons is small throughout. Shortly after switch off, the device is void of free charges and contains only trapped electrons that thermally detrap over time. Therefore, the decay of electron traps in an electron-only device and in a PLED during rest proceeds quite comparable (Note 2, Supporting Information).

Figure 2(b) shows the current evolution of a PLED for varying rest times. First, the device was operated for 300 s and was then rested for 600 s. During rest, electrons detrapped completely and at switch on the current starts at the level of the first run. During the second operation, the same trap dynamics is observed. For shorter rest times (60 s and 10 s), detrapping is not complete and the current starts at a lower level. In these cases, however, the current declines below the level of the preceding measurements, because electron trap formation is not finished after a run-time of 300 s. Finally, the device again fully recovers after a rest time of 900 s. The evolving trap density during an operation time of 300 s in the PLED and the electron-only device is the same (Note 1, Supporting Information); however, as explained above, the current decay in the PLED is much smaller.

These measurements indicate that electron trap formation is fully reversible, and that after thermal detrapping traps disintegrate. We operated a PLED for 1200 s and measured the current recovery during rest (Figure 2(c)). The current recovery kinetics depends on the thermal detrapping rate, from which the trap depth (*E*_*t*_ = 0.67 eV) was derived (Note 2, Supporting Information).

In contrast to the reversible formation of electron traps in electron-only devices, PLEDs irreversibly degrade at longer driving times. When operating a PLED for longer times, the current continuously decreased (Figure 2(d)). This is due to the formation of hole traps [4,16]. Hole traps grow over orders of magnitude and dictate the long-term stability of PLEDs. There is no clear indication in the PLED current trend when electron-trap formation has stopped and when hole traps start to dominate. By assuming that hole traps do not recover, we can estimate when electron-trap formation has finished. After a storage time of 144 h, the current recovered to a value of 8.7 mA cm^−2^ ((i), Figure 2(d)). This current recovery is due to decay of the reversible electron traps and corresponds to the initial current decay when electron traps form ((ii), Figure 2(d)). Thereby, we find that the electron trap formation has finished after 4600 s, with an average value of (3500 ± 1000) s from several measurements. This is in good agreement with the electron trap formation time of 4200 s found for electron-only devices (Figure 1).

We think that these results have implications for future experiments involving current density vs. voltage (*J-V*) characteristics, from which trap energies and densities in general are directly extracted [3,6,8]. If a material is studied where the fraction of slowly evolving electron traps is present, the results of a *J-V* scan depend on the chosen voltage scan rate. If the scan rate is fast, not many traps form during the measurement and the current is much higher than when the scan rate is slow. Therefore, to fully capture the slow electron trap density in a *J-V* measurement, a very slow scan rate must be chosen, or a constant stress bias should be applied before the actual *J-V* scan, during which electron traps develop. Similar arguments apply to hysteresis measurements, e.g. a forward *J-V* scan that is followed by a backward scan [19], which we detail in the Note 3 of the Supporting Information.

## 4. Discussion

We examine the assumption that electron traps form via reduction of an encounter complex between diffusing precursor species. To be specific, we assign the precursor particles to water and oxygen. The reduced water-oxygen complex is an ion-dipole complex and assumed to be stable, but after detrapping, the neutral complex is weakly bound and the trap components separate again via diffusion [20]. We suppose that the kinetics of trap formation reveals details of the trap-formation mechanism. Other potential effects that might explain the slow experimental current trends have been excluded before, such as temperature variations or redistribution of ionic impurities and trapped charges [14].

**Scheme 1.**
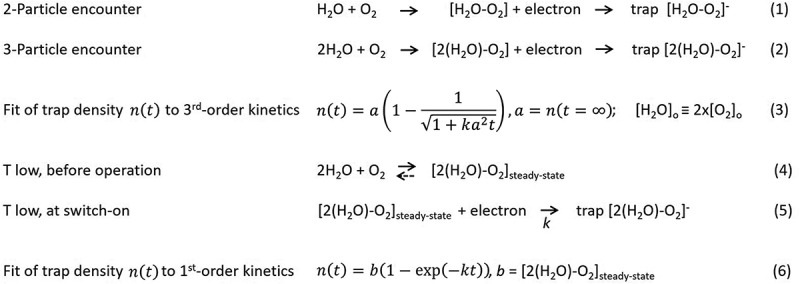
Trap formation via electron trapping of encounter complexes between diffusing water and oxygen molecules.

We ran kinetic Monte Carlo simulations [21] to assess the trap-formation timescale when a trap forms via an encounter between two particles (Scheme 1, Equation(1)). The simulation box was composed of a three-dimensional lattice with 850,000 sites and at the beginning, 85 sites were randomly chosen as oxygen molecules, another 85 as water. Diffusion coefficients for oxygen and water in SY were estimated from the literature (Note 4, Supporting Information). In addition, 0.2% of the total sites were filled with electrons. With these parameters, we mimic the situation in the actual device, with a density of polymer-repeating units of ≈1 × 10^21^ cm^−3^, a total trap density of ≈ 1 × 10^17^ cm^−3^, and a free electron density during operation of ≈2 × 10^18^ cm^−3^. During simulation, oxygen and water could hop to neighboring empty sites, while electrons were kept fixed in their positions. When an electron, a water and an oxygen molecule were within a capture radius of 5 nm, a trap was formed and the involved sites were kept frozen throughout the remainder of the simulation. The simulation continued until no more oxygen and water were free, i.e. until 85 traps have formed.

**Figure 3.**
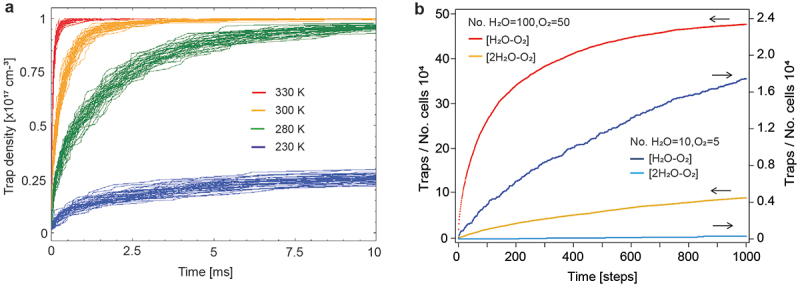
Encounter probability from kinetic monte carlo simulations. (a) Trap formation in a three-dimensional lattice via an encounter between two precursor trap particles. Simulation parameters are detailed in the text and were chosen to mimic the situation in the actual device. (b) Comparison of the encounter probability between two and three particles on a two-dimensional lattice. The lattice was composed of 10^4^ cells and simulations were performed assuming a total trap density of 50 per 10^4^ lattice sites (red, yellow curves) or 5 per 10^4^ lattice sites (dark blue, light blue curves), respectively.

Figure 3(a) displays the simulated trap formation vs. time for temperatures between 230 K and 330 K. Results show that trap formation involving only two particles is very fast. Trap formation at 280 K is finished after 10 ms and is even faster at higher temperatures. This is around five orders of magnitude faster than the experimental timescale for trap formation. The simulated timescale does not depend on the limited size of the lattice dimensions and does not change substantially when the precursor densities, the capture radius or the particle diffusion coefficients are changed over a wide range (Note 4, Supporting Information). We conclude that electron traps do not form via a two-particle encounter.

Due to computational constraints, similar simulations involving three particles (Scheme 1, Equation (2)) were not possible for a three-dimensional lattice. Thus, we resorted to generic kinetic Monte Carlo simulations on a two-dimensional lattice to compare the encounter probability between two and three particles (Note 4, Supporting Information). Experimentally, the kinetics for trap formation does not depend on the current density (Figure S1, Supporting Information). This implies that the lifetime of an encounter complex is long enough such that trapping is faster than complex dissociation and for the current densities applied, every formed complex is trapped by an electron. Therefore, the encounter probability directly corresponds to the probability for trap formation. The simulated trap density dynamics involving three particles depends strongly on the initial particle density, and for low densities as in the experiment, the probability for an encounter between three particles is much lower than for two particles (Figure 3(b)). These simulations confirm the intuitive guess that two diffusing particles encounter rapidly, but that for a dilute system the probability that three particles meet is orders of magnitudes lower [22].

**Figure 4.**
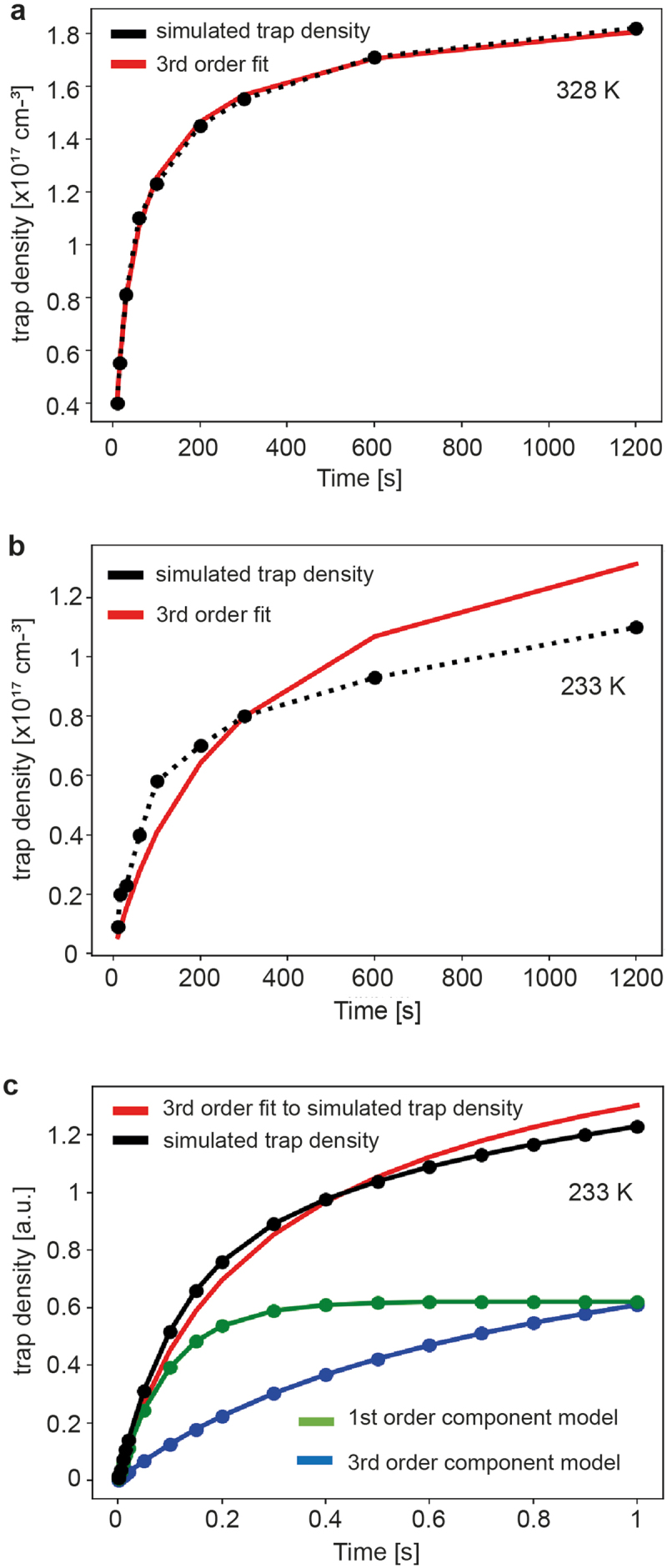
Trap kinetics in electron-only devices. (a) Fit of the simulated trap density at 328 K (data from Figure 1(b)) to a 3^rd^-order kinetics. (b) Fit of the simulated trap density at 233 K (data from Figure 1(b)) to a 3^rd^-order kinetics. (c) Model simulation to demonstrate that the trap density at 233 K can be expressed as a superposition of a slow 3^rd^-order kinetic component and a fast 1^st^-order component.

Figure 4(a) shows a 3^rd^-order kinetic fit to the trap density *n(t)* (data from Figure 1(b)). We fitted the trend with the free fit parameters *a = n(t = ∞)* and *k* (Scheme 1, Equation (3)) [23]. We obtained for the final trap density *n(t = ∞)* = 2.01 × 10^17^ cm^−3^, in good agreement with the simulated value *n(t = 1200 s)* = 1.82 × 10^17^ cm^−3^. The fit is surprisingly good (R^2^ = 0.999), and the trap density does neither follow a clear 2^nd^-order nor a 1^st^-order reaction kinetics (Figure S12, Supporting Information).

For the analysis of the trap kinetics at low temperature (233 K, Figure 4(b)) we adopted the value *n(t = ∞)* = 2.01 × 10^17^ cm^−3^ evaluated at 328 K, because the total trap density is independent of temperature; however, other quantities are temperature-dependent. When lowering the temperature, the diffusion of water and oxygen decreases by orders of magnitude, which lowers the probability that three particles encounter and retards trap formation. It can be seen that the trap dynamics does not follow a 3^rd^-order kinetics anymore; at early times, trap formation is faster than assumed, but at longer times the rate falls below the trend of a 3^rd^-order kinetics.

The difference in trapping kinetics between 328 K and 233 K can be explained by assuming that at low temperature a fraction of the triple-encounter complex, [2(H_2_O)-O_2_]_steady-state_, builds up in the device already before operation (Scheme 1, Equation (4)). At turn-on, this part is trapped rapidly following a 1^st^-order kinetics (Scheme 1, Equation (5)). After that, trap formation is again determined by the probability that three slowly diffusing particles form an encounter complex, which follows a 3^rd^-order kinetics.

When cooling down from 328 K to 233 K, the thermal energy changes by almost 30%. By setting the total concentration of water and oxygen in the active layer to ≈6 × 10^17^ cm^−3^, adopting for the binding energy of [2(H_2_O)-O_2_] in the polymer matrix the calculated gas phase value of 180 meV [24], and estimating [2(H_2_O)-O_2_]_steady-state_ at 233 K to ≈3 × 10^16^ cm^−3^ from the instantaneous trap density rise at turn-on (Figure 4(b)), it follows that [2(H_2_O)-O_2_]_steady-state_ at 328 K is ≈3 × 10^15^ cm^−3^. Such a low trap concentration does not result in a noticeable current decay, which we confirmed by simulation, and explains why the trap evolution at 328 K follows a clear 3^rd^-order kinetics.

Figure 4(c) illustrates with a model simulation the described situation at low temperature. We assumed that the total trap density (black line) is composed of a fast 1^st^-order component (green) and a slow 3^rd^-order component (blue). The total trap density was then fitted to a 3^rd^-order kinetics (red). The fit replicates the essential features observed in Figure 4(b), namely that in the beginning, the actual trap density develops faster than the assumed overall 3^rd^-order kinetics, but at later times the trap evolution is slower.

Inspired by an experiment performed in reference [2], we finally conducted drying experiments to further lower the water content in our samples. Therefore, devices were stored in a desiccator in the glovebox near a powder of the strong desiccant P_4_O_10_ for several days, while reference devices were stored in the same glovebox but away from the desiccant. Measurements showed that the current decay of dried samples was indeed smaller than the decay of non-dried samples (Figure S13, Supporting Information) and we estimate that the water content in the dried material was reduced by ≈20%. We take this as an indication that water is indeed involved in the process of slow electron trap formation.

## 5. Conclusions

In conclusion, we show that universal electron traps in semiconducting polymers are not permanently present, and we propose that they slowly form via a triple encounter between precursor particles, with [2(H_2_O)-O_2_] as a likely candidate. After detrapping, the complex disintegrates and the trap components separate again via diffusion. Therefore, the formation of universal electron traps is a dynamical process that is fully reversible. Further support for this electron trap formation scenario requires the direct detection and quantification of the proposed intermediate species, which is, however, difficult because of the small water and oxygen concentrations present in the material. While our results hopefully resolve an intricate scientific puzzle, they do not deliver new insights of how water and oxygen can be effectively removed from the sample, or how the detrimental effects of electron traps can be further minimized. Because the effect of the first fractions of traps on the device performance decay is very large, it is necessary to remove water and oxygen sample completely, which is difficult. Several practicable techniques by which the operational stability of polymer electronic devices is improved have already been mentioned, including additive-induced trap removal [2,8], trap filling by doping [25], or trap dilution [26]. From an experimental perspective, we remark that the voltage scan rate used in *J-V* measurements is an important parameter. Indeed, because trap filling of permanent traps is finished after ≈200 µs, this parameter has probably not been considered as important so far. However, when *J-V* measurements are performed on samples involving slow electron trap formation and the scan rate is comparable to the trap formation rate, then traps form during the voltage scan, which influences the analysis.

## Supplementary Material

Supplemental Material
