# Supplementary material for: Formation of electron traps in semiconducting polymers via a slow triple-encounter between trap precursor particles
Source: Sci Technol Adv Mater. 2024 Jan 31;25(1):2312148. doi: 10.1080/14686996.2024.2312148 (PMC10868412; doi:10.1080/14686996.2024.2312148)
Supplement: Supplemental Material [file TSTA_A_2312148_SM0344.docx]

**Supporting Information**

**Formation of electron traps in semiconducting polymers via a slow triple-encounter between trap precursor particles**

Mohammad Sedghi, Camilla Vael, Wei-Hsu Hu, Michael Bauer, Daniele Padula, Alessandro Landi, Mirko Lukovic, Matthias Diethlem, Gert-Jan Wetzelaer, Paul W. M. Blom, Frank Nüesch, Roland Hany*

*Empa, Swiss Federal Laboratories for Materials Science and Technology, Laboratory for Functional Polymers, 8600 Dübendorf, Switzerland. roland.hany@empa.ch


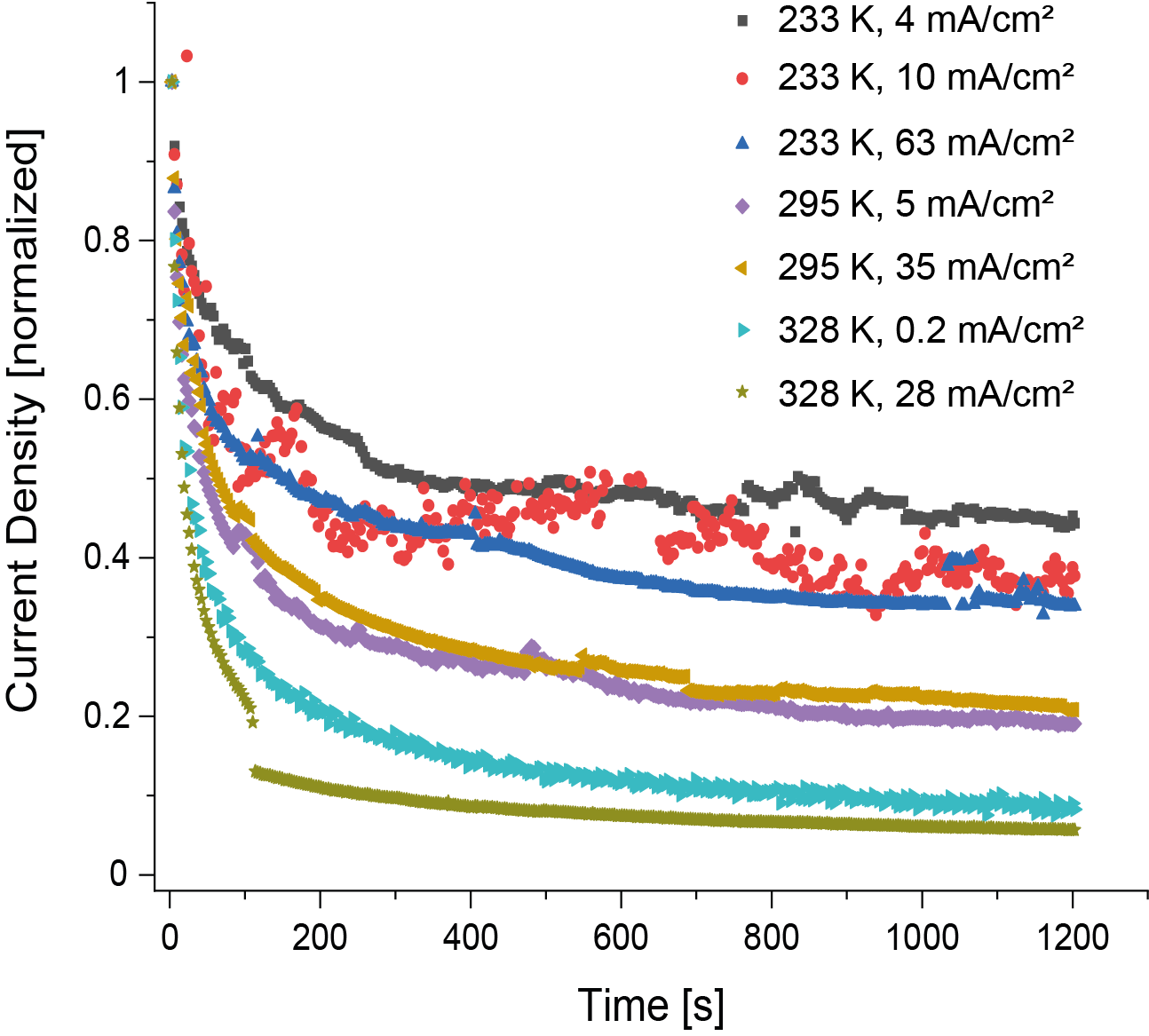


**Figure S1.** Current trends for electron-only devices at different temperatures and current densities applied. At a given temperature, the dynamics of the current decay is independent of the current density. For permanent traps, trap filling would depend on the current density and the time for trap filling decreases when more electrons flow through the device [1]. However, for any current density applied, trap filling of permanent traps would be much faster than the sampling rate between individual points (0.3 s^-1^) of the experiment. The observation that the current decay is independent of the current density indicates that if an encounter between precursor trap species occurs, the lifetime of the complex is long enough such that trapping is faster than complex dissociation and within the current densities measured, all available complexes are charged. This holds true for every temperature.

**Note 1, Simulation of trap dynamics**

Electrical drift-diffusion simulations were performed with Setfos 5.2 from Fluxim AG.

| **Parameter** | **Value** | **Source** |
| --- | --- | --- |
| Thickness SY | 80 nm | Measured with profilometer |
| LUMO SY | 2.95 eV | Reference [2] |
| HOMO SY | 5.45 eV | Reference [2] |
| Dielectric constant SY | 3 | Reference [3] |
| Effective density of states | 1 x 10^27^ 1/m^3^ | Reference [1] |
| Work function PEDOT:PSS | 5.35 eV | Chosen to have a barrier for hole injection of 0.1 eV |
| Work function Al anode | 4.08 eV | Setfos manual |
| Work function Ca | 3.05 eV, equal to 1 x 10^19^ electrons cm^-3^ | Setfos manual, chosen to have a barrier for electron injection of 0.1 eV |
| Electron trap energy vs. vacuum | 3.6 eV | Reference [4] |

**a) Electron-only devices**, ITO/Al 20 nm/SY/Ca 10 nm/Al 70 nm

Relevant input parameters in the EGD model are the width of the Gaussian density of states for the SY HOMO / LUMO levels (σ) and the electron trap distribution (σ_t_), as well as the mobility at zero field and T = ∞ (µ_0_). For SY, these parameters were adopted from reference [5,6]: σ = 0.14 eV, σ_t_ = 0.1 eV, µ_o_ = 1.1 x 10^-6^ m^2^ V^-1^ s^-1^.

Before the actual current decay measurement over time was performed, a constant current of 10 mA cm^-2^ was applied at each temperature to read out the required operating voltage. During this time of around 3 s, already some electron traps formed. Therefore, the starting currents for the subsequent measurements were not exactly 10 mA cm^-2^, but were slightly lower. In Figure 1, main text, we show the normalized (to 10 mA cm^-2^) current decay trends, but the experimental currents were simulated.

In a first simulation step, the trap density was set to 0 and the initial current was adjusted by slightly varying the voltage and µ_o_(T). For example at 273 K, the initial current of 7.5 mA cm^-2^ was simulated with V_fit_ = 4.4 V and µ_o_(273)_fit_ = 1.2 x 10^-8^ cm^2^ V^-1^ s^-1^, close to V_exp_ = 4.8 V and µ_o_(273)_calc_ = 3.8 x 10^-9^ cm^2^ V^-1^ s^-1^. Fit parameters were then kept constant and traps were added in the simulation to match the measured current decay over time.

The simulation distinguishes between *N* (Figure S2), which is the number of available trap sites, and *n*, the number of trap sites that is actually filled in steady state, which is equivalent to the number of trapped electrons. In Figure 1, main text, we show the simulated *n(t)* values. In our trap formation scenario, *N(t)* is not constant but we can identify *N(t)* with the number of trap precursor encounters that increases with time, *n(t)/N(t)* of which are actually reduced by an electron and form a trap. The influence of a certain value for *N* on the current decay and the ratio *n/N* are not independent of the temperature. For example, *n/N* increases slightly with decreasing temperature; this is because the free electron density increases in the device when lowering the temperature, and a larger fraction of available trap sites is filled. Also, *n/N* decreases when *N* increases; again, this is because the ratio between the free electron density and *N* decreases. These trends affect the exact number of the simulated trap site density, but they cannot explain the observation that the current decay, and therefore the dynamics of trap formation, strongly increases with increasing temperature. For example, when *N(328 K, 1200 s)* = 3 x 10^17^ cm^-3^ is used as simulation input at 233 K, the calculated current after 1200 s is 0.60 mA cm^-2^ (close to the measured current 0.68 mA cm^-2^ at 328 K), but the experimental current at 233 K after 1200 s was 4 mA cm^-2^.

By setting *N(t=0)* = 0 we simulate those electron traps that develop slowly over time and neglect any permanent electron traps that are already present in the material when the device is switched on. We found before that a fraction of around 1 x 10^17^ cm^-3^ electron traps is present in pristine SY for which charge trapping takes around 200 µs [7]. The simulated slow electron trap dynamics does not change when electron traps in the pristine material are included. For example, when setting *N(t=0)* = 1 x 10^17^ cm^-3^, the simulated electron trap dynamics does not change but the total electron trap density increases by ~8 x 10^16^ cm^-3^, and this value is independent of time and temperature.


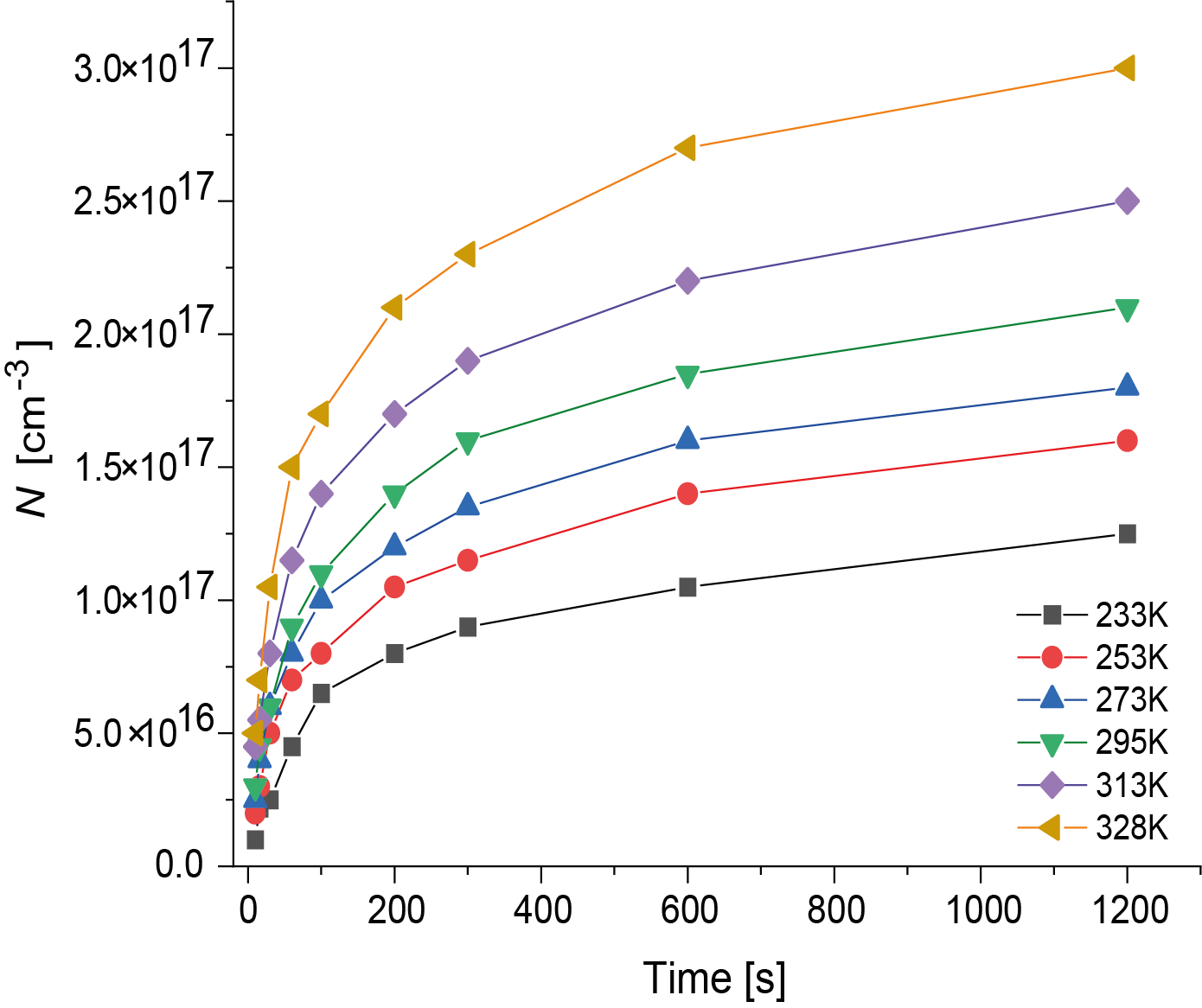


**Figure S2.** Simulated number of the total trap site density over time, *N(t)*. Each symbol is the result of one simulation, in which *N* was added to match the experimental current. *N(t)* and *n(t)* values from Figure 1, main text, show the same trend.

As described in the main text, we simulated the trap density with the trap depth of 0.65 eV. We should note that this choice is relatively insensitive to the outcome of the result within a range of ≈ ±0.1 eV for the following reason: As explained above the simulation distinguishes between *N*, which is the number of available traps, and *n*, which is the number of traps that are actually filled in steady state. Taking the trap density at 328 K after 1200 s as an example, we simulated *N(t=1200 s)* = 3 x 10^17^ cm^-3^, *n(t=1200)* = 1.82 x 10^17^ cm^-3^, *E_t_* = 0.65 eV. If we change *E_t_* to 0.75 eV or 0.55 eV, respectively, then *N(t=1200 s)* changes substantially to 2.1 x 10^17^ cm^-3^ (*E_t_* = 0.75 eV) or 6.5 x 10^17^ cm^-3^ (*E_t_* = 0.55 eV) but *n(t=1200 s)* does not change. *n* is the relevant number of traps that determines the measured current level, while *N* is the required number of trap states such that *n* is reached. If the trap depth is increased, than thermal detrapping decreases and a larger fraction of *N* is actually filled. On the other hand, for a smaller *E_t_* thermal detrapping gets more important, and a larger *N* is required such that *n* traps are filled.

We can exclude, however, that shallow electron traps are involved in the slow trap formation process. For an assumed *E_t_* = 0.3 eV, the required simulated number of *N*, which corresponds in our case to the water and oxygen impurity content in the material, amounts to *N* = 6 x 10^19^ cm^-3^. This is exceedingly large because it would mean that the water and oxygen content in the polymer is around 2%.

Finally, we noticed in the main text that the trap filling rate of permanent traps depends little on temperature. The average simulated electron mobility, and thereby the capture rate for electrons [1], decreases when lowering the temperature from 328 K to 233 K, but only by a factor of ≈3. This means that trap filling at lower temperatures slows down. At the same time, however, the average electron density in the device increases by about 30%, which results in faster trap filling. These two effect compensate each other, making filling of permanent traps largely independent of temperature. Small deviations from this statement are not relevant in the context of our results, because filling of permanent traps will be finished at any temperature after a few hundred µs, which is orders of magnitudes faster than our experimental trap evolution time of many minutes.

**b) PLEDs**, ITO/PEDOT:PSS 40 nm/SY/Ca 10 nm/Al 70 nm

For the device simulation at 295 K, the electron trap density before operation was set to zero. The mobilities of electrons and holes in the PLED were described by the EGD model and electron traps were introduced as Gaussian traps with a depth of 0.65 eV below the LUMO, with a distribution width of 0.1 eV. The simulations included Langevin recombination of free electrons and holes, and trap-assisted recombination of trapped electrons with free holes, with the capture coefficients for electrons and holes given by *C_n(p)_* = *qµ_n(p)_*/*ε*. The simulated current density of the PLED was first set to 10 mA cm^-2^, and the trap density was increased to account for the current decrease as a function of time. The time-dependent trap density is given in the table below. As the PLED current is dominated by the (trap-free) hole transport, the total current is less influenced by the generation of electron traps, implying that the PLED current is more stable than the electron current over time (Note 2, Supporting Information). However, the observed decrease in PLED current could be described with a similar trap-density growth as observed for the electron-only devices, also considering that the average (from 8 experiments) PLED current after an operation time of 1200 s arrived at (9.1 ± 0.2) mA cm^-2^, which corresponds to a current decay of (100 ± 20)%. This shows that electron formation in both PLEDs and electron-only devices are consistent.

|  | PLED | Electron-only device |
| --- | --- | --- |
| Time [s] | *N(t)*  [x10^16^ /cm^3^] | *N(t)*  [x10^16^ /cm^3^] |
| 10 | 4 | 3 |
| 30 | 6 | 6 |
| 60 | 7 | 9 |
| 100 | 8 | 11 |
| 200 | 9.5 | 14 |
| 300 | 11 | 16 |
| 600 | 13 | 18.5 |
| 1200 | 16 | 21 |

**Note 2, Trap-related processes in a PLED during operation and at rest**

During operation of a PLED, free holes recombine with free electrons (Langevin recombination) and trapped electrons (SRH recombination). Because of SRH recombination, a fraction of trapped electrons is constantly depleted during operation, such that the number *n* of trapped electrons is considerably smaller than the total trap site density present (*N*). This is in contrast to an electron-only device, where the density of trapped electrons is approximately equal to the total trap site density. When the density of immobile trapped electrons increases strongly, the flow of free electrons decreases. This is similar to the electron-only device. However, while in the electron-only device the overall current then drops to zero, in the PLED a substantial current still remains. This current is entirely due to injected holes that recombine with trapped electrons. For example, for a trap site density of 5 x 10^17^ cm^-3^, the simulated PLED current is still at 5.7 mA cm^-2^, and remains at a value of ≈3.6 mA cm^-2^ for trap densities >>10^19^ cm^-3^. Therefore, the current in a PLED is referred to as "hole dominated". The outcome is that the current decay in a PLED due to the presence of electrons traps is much less than in an electron-only device, which makes the simulation of electron traps in PLEDs more difficult.

Less obvious are the processes inside a PLED when the device is switched off. A reasonable assumption is that free holes recombine rapidly with free and trapped electrons and that the device is free of electronic charge after a few microseconds. However, this turns out to be not the case (Figure S3).


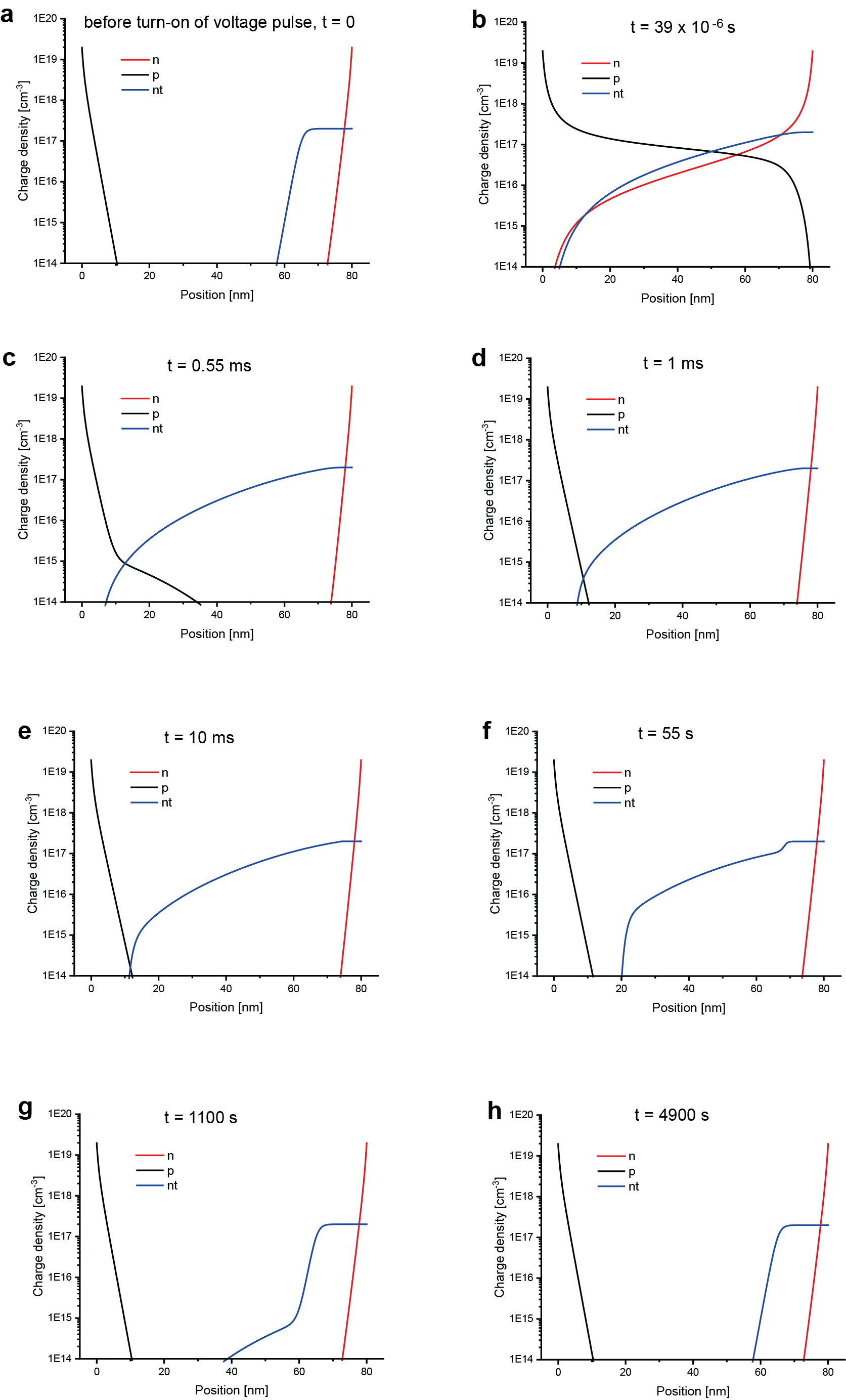


**Figure S3.** Transient simulation of a PLED with an assumed trap density of 2 x 10^17^ cm^-3^ and a trap energy of *E_t_* = 0.65 eV. (a) At t = 0, a voltage pulse of 3.7 V was applied for 500 µs, after which the voltage was turned off and set to 0 V. (b) Steady state was reached after an operation time of ≈39 µs. (c) t = 0.55 ms is the first simulation point after switch off. Holes and free electrons recombine within a few µs when the voltage is set to 0 V, or are extracted rapidly at the electrodes. A small fraction of holes recombines with trapped electrons via SRH recombination. (d) and (e) After 10 ms, SRH recombination is terminated, the density of free holes and electrons has dropped to ≈0, and trapped electrons remain inside the device. (f – h) Electron traps detrap via thermal emission, which is terminated after ≈4900 s, when the device situation before the voltage pulse is restored. The simulation shows that the decay of electron traps in an electron-only device and a PLED during rest proceeds quite comparable.

The existence of trapped electrons in the PLED after switch off can be demonstrated by irradiating the device with light below the bandgap of SY during relaxation, which photo-excites and releases trapped electrons into the conduction band of the active material (Figure S4).

**
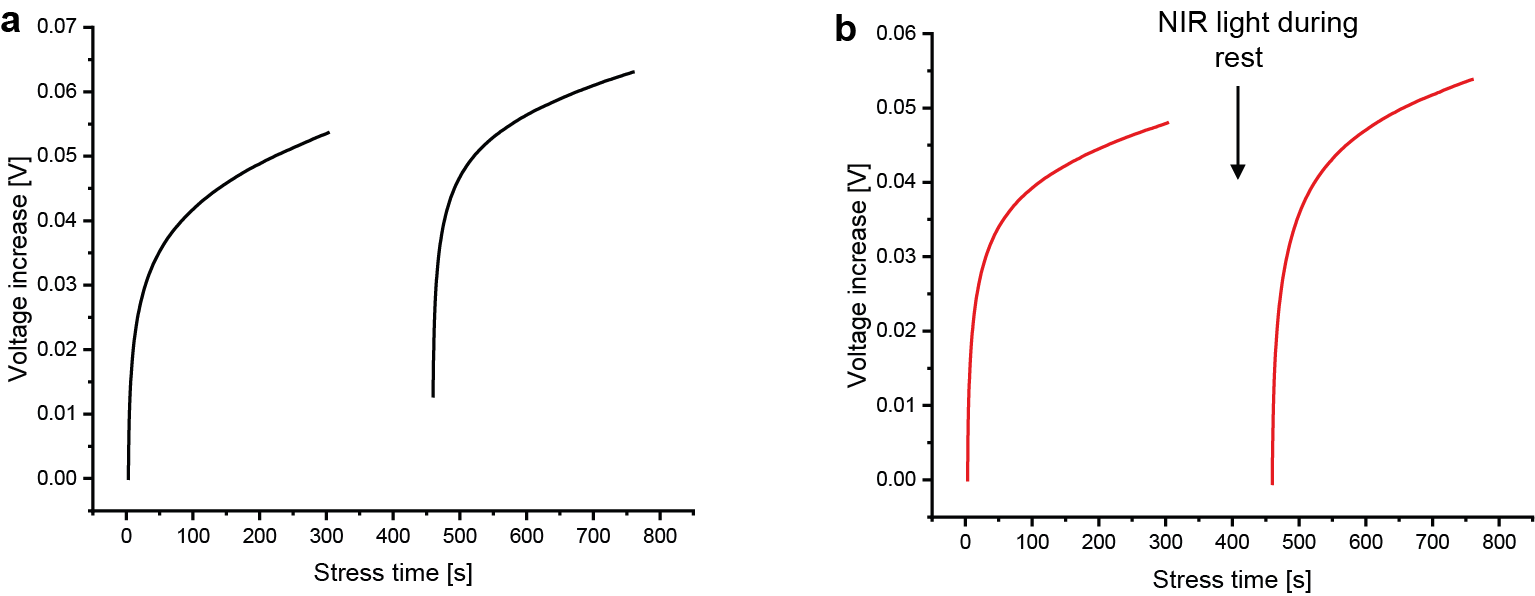
**

**Figure S4.** Increase in voltage for a device at 230 K during two stressing cycles at 10 mA cm^-2^ for 300 s with a rest time of 160 s at 0 V in between. (a) The device was kept in the dark during rest. (b) The device was illuminated with light of wavelength 855 nm during rest. In (a), the voltage of the second stressing pulse started at a higher value than the voltage of the first stressing pulse because electron detrapping is not complete after a rest time of 160 s. In (b), the voltage of the second stressing pulse started at the same value as the voltage of the first stressing pulse. This indicates that NIR light released trapped electrons into the SY conduction band that remained in the device after turn off.

The kinetics of electrons that thermally detrap is given by [7]

with the detrapping rate

where *E_t_* is the trap energy, *LUMO* is the energy of the lowest unoccupied molecular orbital of SY (2.95 eV), *C_n_* is the electron capture rate (5 x 10^-13^ cm^3^ s^-1^), and *N_o_* = 10^27^ m^-3^, which is the result of one state nm^-3^.

After the time *t* = *1/e_n_*, *n(t)* = 0.37 x *n(0)*, which means that 63% of the trapped electrons have thermally detrapped. The detrapping time constant *t* = 660 s from Figure 2c, main text, indicates *E_t_* ≈ 0.67 eV. The time constant sensitively depends on the trap depth, and *t* = 44 s for *E_t_* = 0.6 eV, or *t* = 16600 s for *E_t_* = 0.75 eV, respectively.

**Note 3, *J-V* scans and hysteresis in the presence of slow electron trap formation**


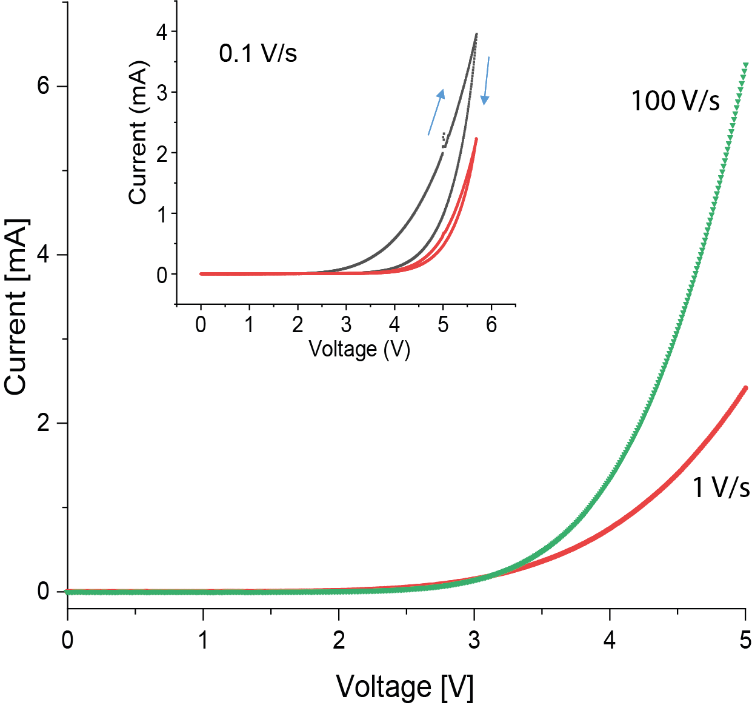


**Figure S5.** Current-voltage (*J-V*) scans for pristine electron-only devices with scan rates of 1 V s^-1^ and 100 V s^-1^. Insert: For a pristine (black lines) electron-only device, the voltage was scanned from 0 V to 5.5 V and back with a scan rate of 0.1 V s^-1^. Then, the device was operated at 4.3 V for 30 minutes, and the forward and backward scan was repeated (red lines).

During a *J-V* sweep, the voltage is increased gradually and the current is measured. A typical experimental voltage scan rate is 0.1 V s^-1^. Because our presumption is that filling of existing electron traps is fast and finished after ≈200 µs, the device is in constant equilibrium during the scan. One effect to consider for permanent traps is that the number of available traps (*N*) that is actually filled (*n*) increases when the voltage increases. Consider for permanent traps an up-scan followed by a back-scan (insert of Figure S5). During the up-scan *n* increases continuously, and because thermal detrapping is slow (for a trap depth of *E_t_* ≈ 0.65 eV), *n* at a given voltage during the back-scan is larger than during the up-scan, which results in a decrease of the current. The outcome is a hysteresis in the measured current, with the current during the up-scan being larger than the current during the back-scan.

This source of hysteresis in the *J-V* characteristics, however, does not explain the observation that the current during the hysteresis measurement after 30 minutes of operation is smaller than the current of the pristine device (Figure S5, insert). Furthermore, it does also not explain why the current during the up-scan strongly depends on the chosen voltage scan rate (Figure S5, main). We ascribe these effects to the formation of electron traps during the measurement.

If the overall time for the scan is only 1/20 s (scan rate 100 V s^-1^) not many traps form during the scan and the current is much higher than when a lower scan rate is chosen (1 V s^-1^). It is also clear that the *J-V* scan strongly depends on whether a pristine or a stressed device is measured. Trap formation during the scan superimposes with increasing charge trapping of permanent electron traps at higher voltages.

We can illustrate this scenario with simulations of electron-only devices including the EGD model (Figure S6), where we assume a trap depth of *E_t_* = 0.65 eV. When adding a constant trap density *N* = 1 x 10^17^ cm^-3^ (red simulation points), the current decreases compared to the trap-free simulation (black simulation points). In the simulation, *n* increases at higher voltages, e.g., from 7.6 x 10^16^ cm^-3^ at 2.5 V to 8.6 x 10^16^ cm^-3^ at 6 V. This effect results, as explained above, in a current hysteresis.

We also simulated the current due to the slowly evolving electron traps (green points). We assumed a scan rate of 0.1 V s^-1^ and took *N(t)* as function of time from Figure S2. The simulated current "*N_exp_(t)*" (violet points) would then correspond to the measured current during the up-scan, where *N_exp_(t)* is the sum of *N* = constant plus *N(t)* that forms during the scan. For a higher voltage scan rate than 0.1 V s^-1^, the influence of *N(t)* would be smaller and the current "*N_exp_*" shifts in direction of the red current trend, for a slower scan rate the current would drop below the simulated violet current trend.


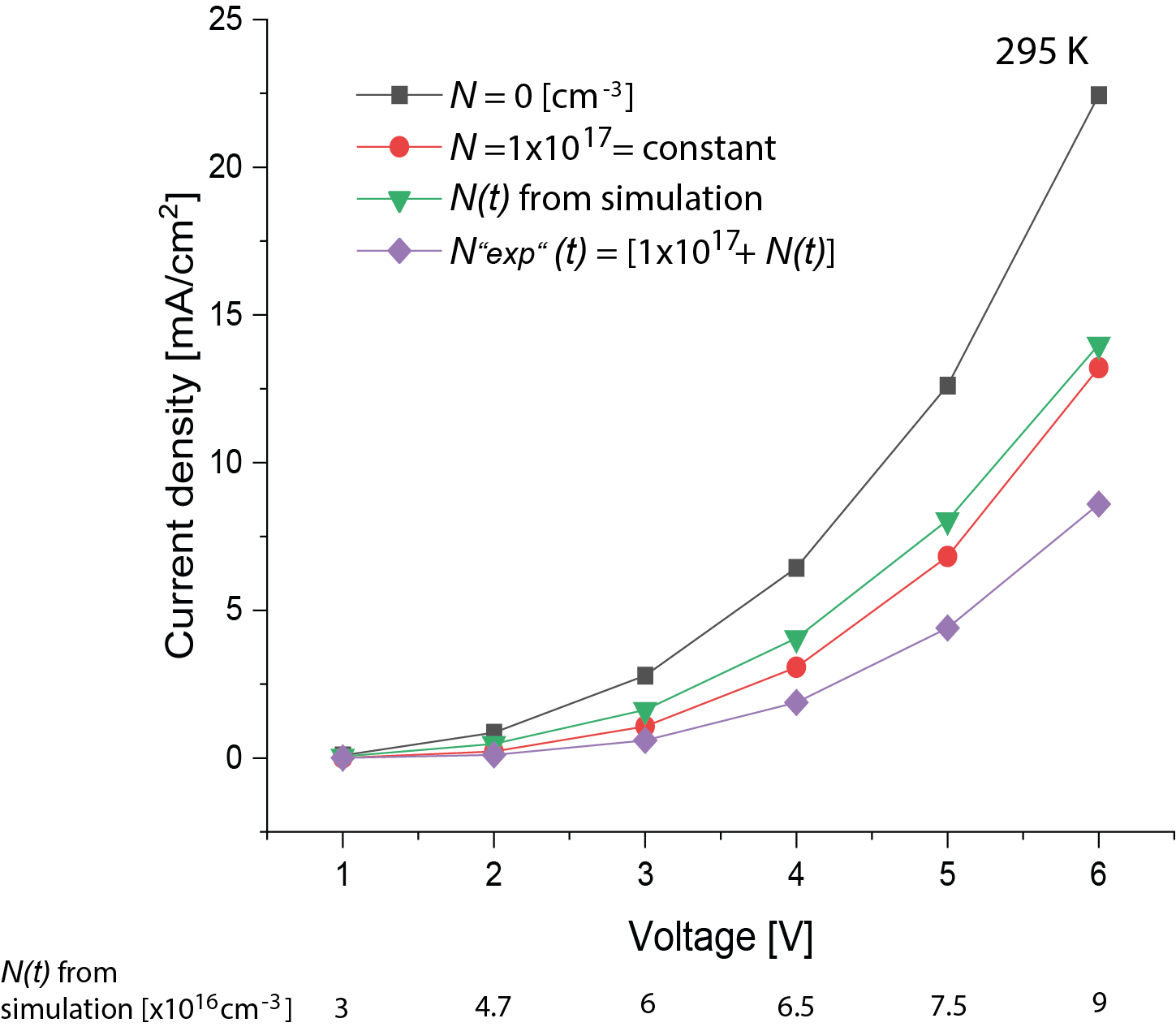


**Figure S6.** Simulated *J-V* trends in the presence of permanent and slowly evolving electron traps.

**Note 4, Kinetic Monte Carlo Simulations**

**a) Simulation details for Figure 3a, main text**

We ran kinetic Monte Carlo simulations with an in-house developed code [8]. The simulation box was composed by a lattice with dimensions (100 x 100 x 85) nm^3^, where 85 nm corresponds to the active layer thickness. Periodic boundary conditions were applied along the *x* and *y* directions, and sites were arranged at a distance of 1 nm from each other, so a total 850000 sites are available. At the beginning, 85 sites were randomly chosen as oxygen molecules, and other 85 as water. These concentrations mimic the situation in the actual device, with a density of polymer-repeating units of ≈ 1 x 10^21^ cm^-3^, and a total trap density of ≈10^17^ cm^-3^. In addition, 0.2% of the total sites were filled with electrons (Figure S7). The latter were also chosen randomly, but with the additional constraint of following a concentration gradient along the *z*-axis to reproduce the simulated electron density in the device. The remaining sites were left empty. Any empty site could be occupied by water and oxygen particles when they diffuse during the simulation, while electrons were kept fixed in their initial positions.

At each time step, each oxygen and water particle could hop to a neighboring empty site. We used estimated diffusion coefficients for water and oxygen to compute diffusion rates $k_{i}$, the probability of a particle moving to any neighboring empty site being proportional to the diffusion rate (see Table below). The simulation time was increased by drawing a random time from the exponential distribution of the total rate constant $k_{tot}=\sum k_{i}$. When an electron, a water molecule and an oxygen molecule were within a 5 nm radius (Figure S8), a trap was formed and the involved sites were kept frozen throughout the simulation. The simulation continues until no more oxygen and water are free, *i.e.* until 85 traps are formed. We did not consider any detrapping mechanism in our simulations.

We ran simulations at 230 K, 280 K, 300 K and 330 K, propagating 40 independent trajectories from the same initial conditions. The effects of different temperatures were taken into account by considering the different diffusion coefficients (and thus different rates).

From the simulations shown in Figure 3a, main text, it can be seen that trap formation between 280 K and 330 K is finished after ≈10 ms. We ran simulations to test the influence of several parameters on the overall timescale for trap formation. When the water density is increased to 4 x 10^17^ cm^-3^, trap formation is faster – as expected – and is finished after ≈1 ms. It is clear that trap formation would slow down if the water density were lowered below 1 x 10^17^ cm^-3^; however, we did not simulate this situation because this does not correspond to our real-world device.

Lowering the diffusion rate of water by two orders of magnitude (from 6 x 10^7^ s^-1^ to 6 x 10^5^ s^-1^) slows down the trap formation. However, trap formation is still finished after 30 ms and thereby orders of magnitudes faster than our experimental trap formation timescale.

For smaller lattice dimensions we found that trap formation is faster and scales approximately quadratically with N (N x N x 85). Due to limited computational resources, we could not run simulations with larger lattice dimensions than (100 x 100 x 85) but we think this dimension is sufficient to mimic our real-world device. In Figure 3a, main text, it can for example be seen that trap formation at 300 K is finished after ≈2 ms. With D = 10^-8^ cm^2^ s^-1^, the typical diffusion length during this time is <L^2^>^1/2^ = (t x 2 x D)^1/2^ ≈ 100 nm, which is of the same order as our lattice dimensions. This means the trap formation timescale would not increase strongly if we were able to further increase the lattice dimensions in the simulation, because trap formation occurs locally in a small partial volume of the total active layer volume.


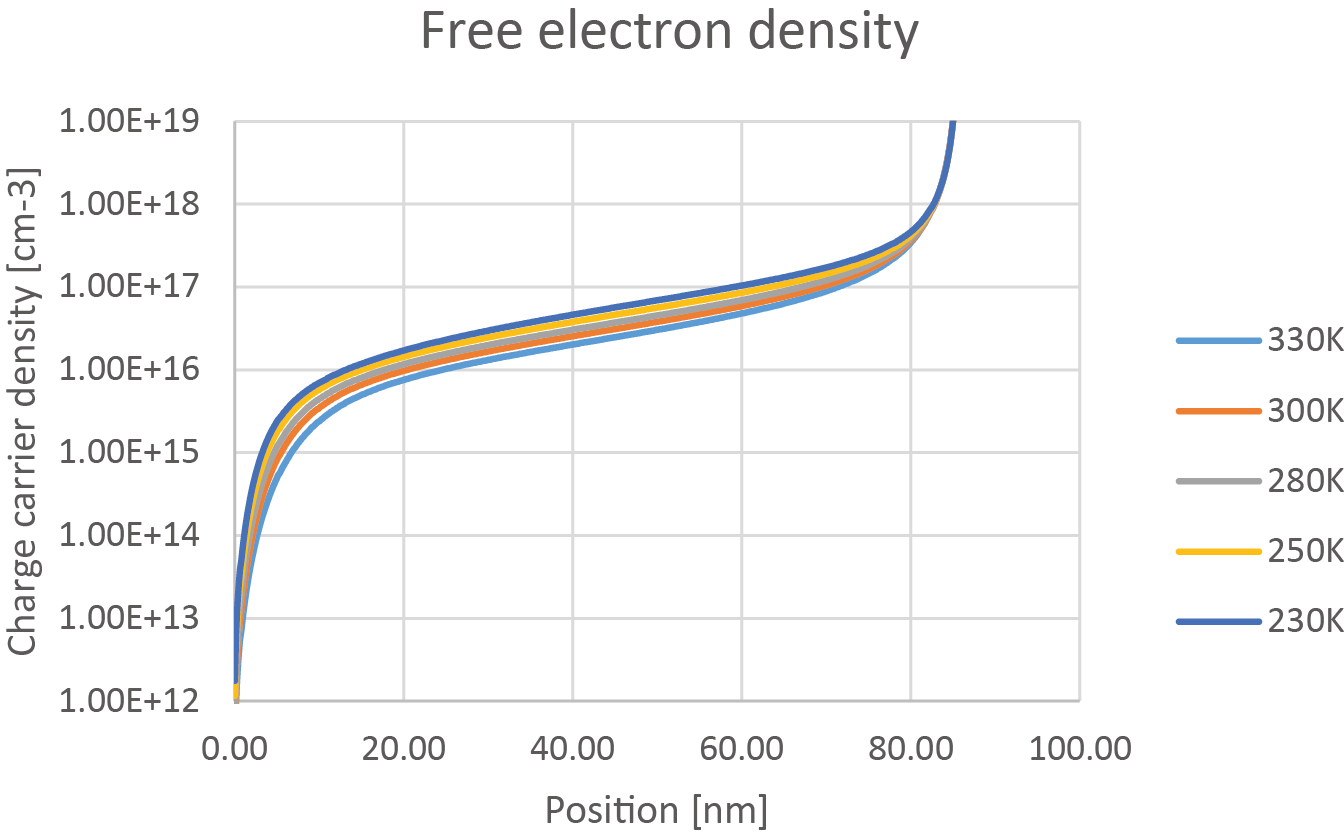


**Figure S7.** The simulated free electron density in steady state is strongly inhomogeneous over the active layer thickness but does not strongly depend on the temperature. For example, at 330 K, the average free electron density is 1.4 x 10^14^ cm^-3^ (0-5 nm), 6.4 x 10^15^ cm^-3^ (5-30 nm) 2.3 x 10^16^ (30-55 nm), 1 x 10^17^ (55-80 nm), and 1.9 x 10^18^ cm^-3^ (80-85 nm). When cooling down to 230 K, these numbers do not change by a factor of more than 2. The total free electron density is ≈2 x 10^18^ cm^-3^, and with a density of polymer-repeating units of ≈ 1 x 10^21^ cm^-3^ it follows that around 0.2% of all sites in the simulation are occupied with an electron.

Diffusion coefficients and rate constants used in the kinetic Monte Carlo simulations.

| *T* / K | H_2_O | | O_2_ | |
| --- | --- | --- | --- | --- |
|  | *D* / cm^2^ s^-1^ | *k* / s^-1^ | *D* / cm^2^ s^-1^ | *k* / s^-1^ |
| 230 | 4E-12 | 2.4E3 | 3E-10 | 1.8E5 |
| 280 | 2E-9 | 1.2E6 | 1E-8 | 6E6 |
| 300 | 1E-8 | 6E6 | 4E-8 | 2.4E7 |
| 330 | 1E-7 | 6E7 | 2E-7 | 1.2E8 |

To our knowledge, the diffusion coefficients (D) as function of temperature for oxygen and water in SY are not known. For a range of glassy commodity polymers (e.g. PMMA, polycarbonate, poly(ethylene-co-norbornene) and semiconducting polymers (P3HT, PCDTBT, PTB-7), the D for oxygen at room temperature is in the range of D(O_2_,298 K) = (1-5)x10^-8^ cm^2^s^-1^, and the activation energy for oxygen diffusion is E_a_ ≈ (360 ± 50) meV [9-13]. With Arrhenius relations from reference [9], we calculated D(O_2_) values for other temperatures, i.e. D(330 K) = 2x10^-7^, D(300 K) = 4x10^-8^, D(280 K) = 1x10^-8^, and D(230 K) = 3x10^-10^ cm^2^s^-1^.

The situation is less homogeneous for the diffusion of water [14-16], also because of the ability of water to form hydrogen bonds; consequently, D(H_2_O) depends strongly on the polymer type. For example for PMMA, D(298 K) = 1x10^-8^, 3x10^-8^, or 3x10^-9^ cm^2^s^-1^ have been measured [17-19]. Also, measured activation energies vary considerably, e.g. E_a_ ≈ 40 kJmol^-1^ for PMMA [18] or polyetherimide [20], ≈51 kJmol^-1^ for epoxy materials [15], or 64 kJmol^-1^ for polyamide [14]. D(H_2_O) at different temperatures was fixed as 1x10^-7^ (330 K), 1x10^-8^ (300 K), 2x10^-9^ (280 K), and 4x10^-12^ cm^2^s^-1^ (230 K)^14^.

**
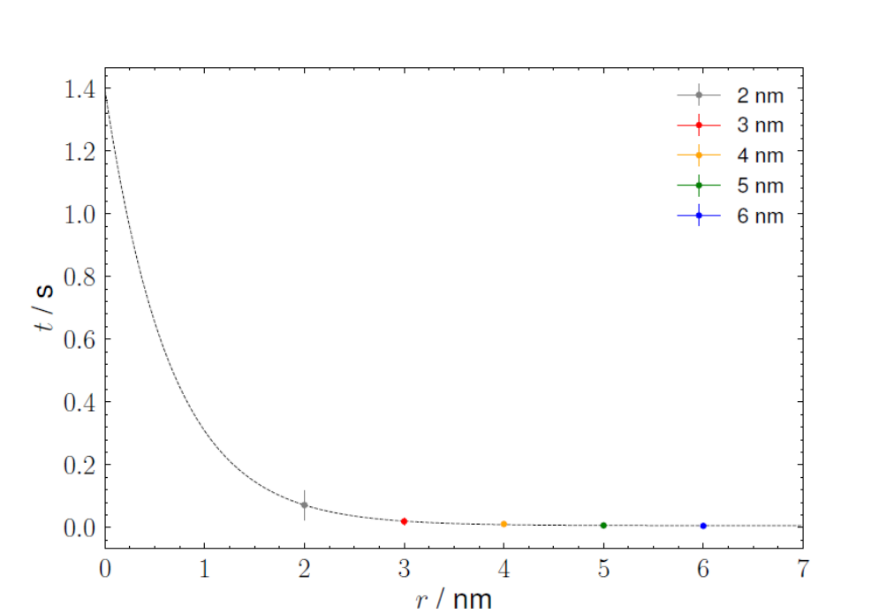
**

**Figure S8.** The capture radius r is defined as the radius within which an encounter between an electron, a water and an oxygen molecule forms a trap. Decreasing r results in an increase of the trap formation time. However, even when extrapolating to r → 0, trap formation does not take more than ≈1 s, which is 3 orders of magnitude faster than the measured trap formation time (at 295 K). The drawn curve is t = c + a x exp(-b x r), with the fitted parameters a = 1.39, b = 1.53, c = 0.01.

**b) Simulation details for Figure 3b, main text**

Simulations were performed on a 2D-lattice composed of 100 x 100 = 10^4^ cells. Each cell can be empty or contains a single O_2_ or H_2_O molecule at a time. The simulation starts from a random configuration of empty, O_2_-occupied and H_2_O-occupied cells. Each cell can contain only one molecule at a time, and in the model, each cell contains four neighboring cells (north, east, south, west). Periodic boundary conditions were used in the simulation, the random walk takes place on the surface of a torus.

The evolution of the system (a time step) consists first of a reaction stage and then a diffusion step. A reaction between three particles occurs and a trap forms if there are at least two H_2_O molecules in the neighborhood of a cell containing an O_2_ molecule, or if there is at least one H_2_O and one O_2_ molecule in the neighborhood of a H_2_O molecule. A reaction between two particles occurs if one O_2_ and one H_2_O molecule occupy neighboring cells.

During diffusion, each particle can make a move to a randomly selected adjacent cell. At every time step, O_2_ and H_2_O make a move with probabilities p(O_2_) and p(H_2_O). For most of the simulations, p(O_2_) = 100 x p(H_2_O) was chosen, but simulation results are largely independent of the chosen probability ratio (see below). During diffusion, a molecule can only move to an empty adjacent cell. If the selected cell is already occupied, the move is not made.


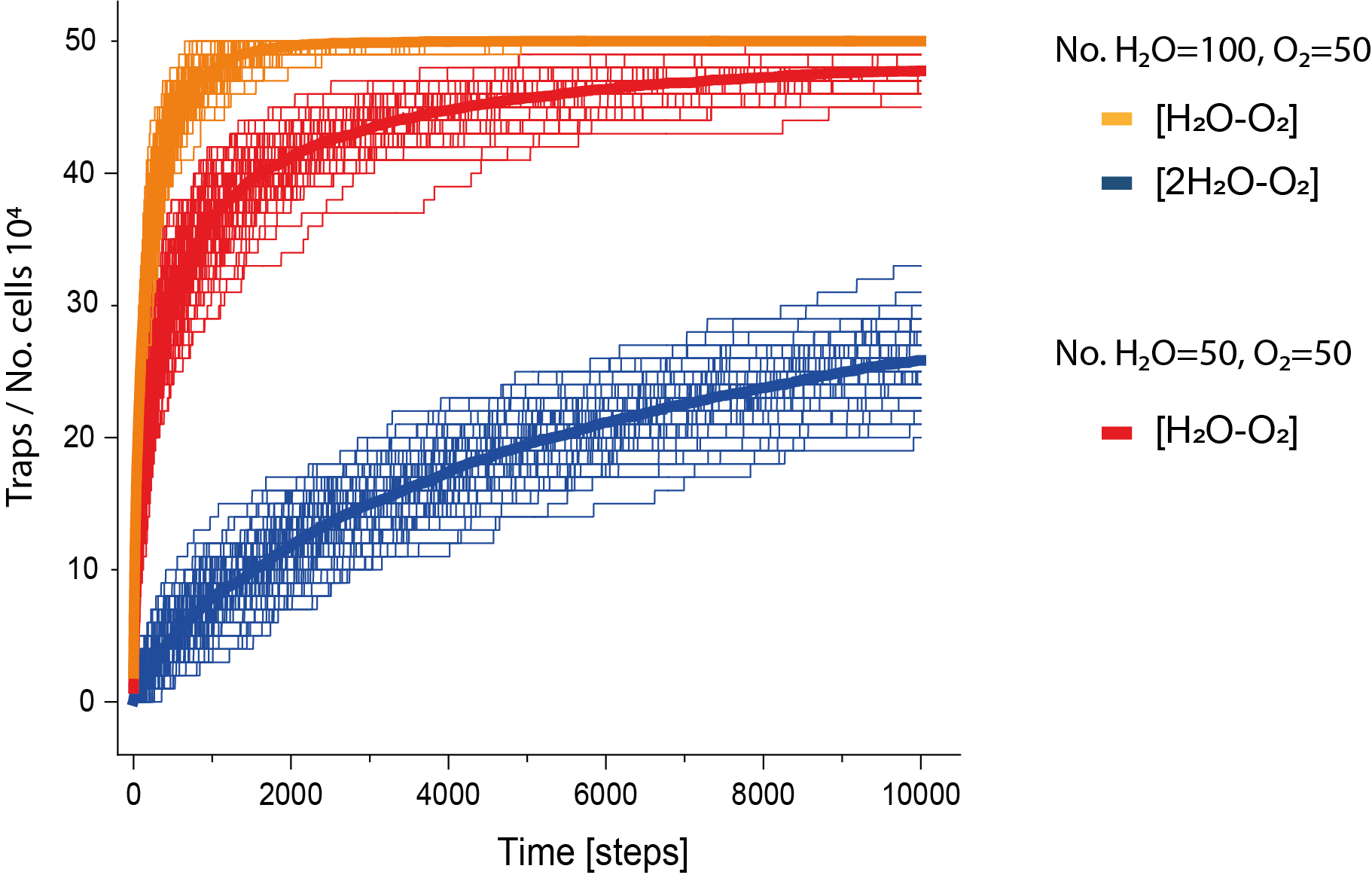


**Figure S9.** Variability, average value and long-term trap evolution in the simulation. In these examples, 0.5% of all cells were occupied with O_2_. Therefore, the maximum trap density is 50 traps/10^4^ lattice sites. Compared to the actual situation in the device where the trap density is in the order of 1 trap per 10^4^ polymer-repeating units, the trap density in these simulations is very high. In the beginning, the probability for trap formation involving 3 particles is substantial. However, as time progresses and the concentrations of H_2_O and O_2_ decrease, the probability for a triple-encounter decreases strongly and the trap number asymptotically approaches the total trap density of 50.


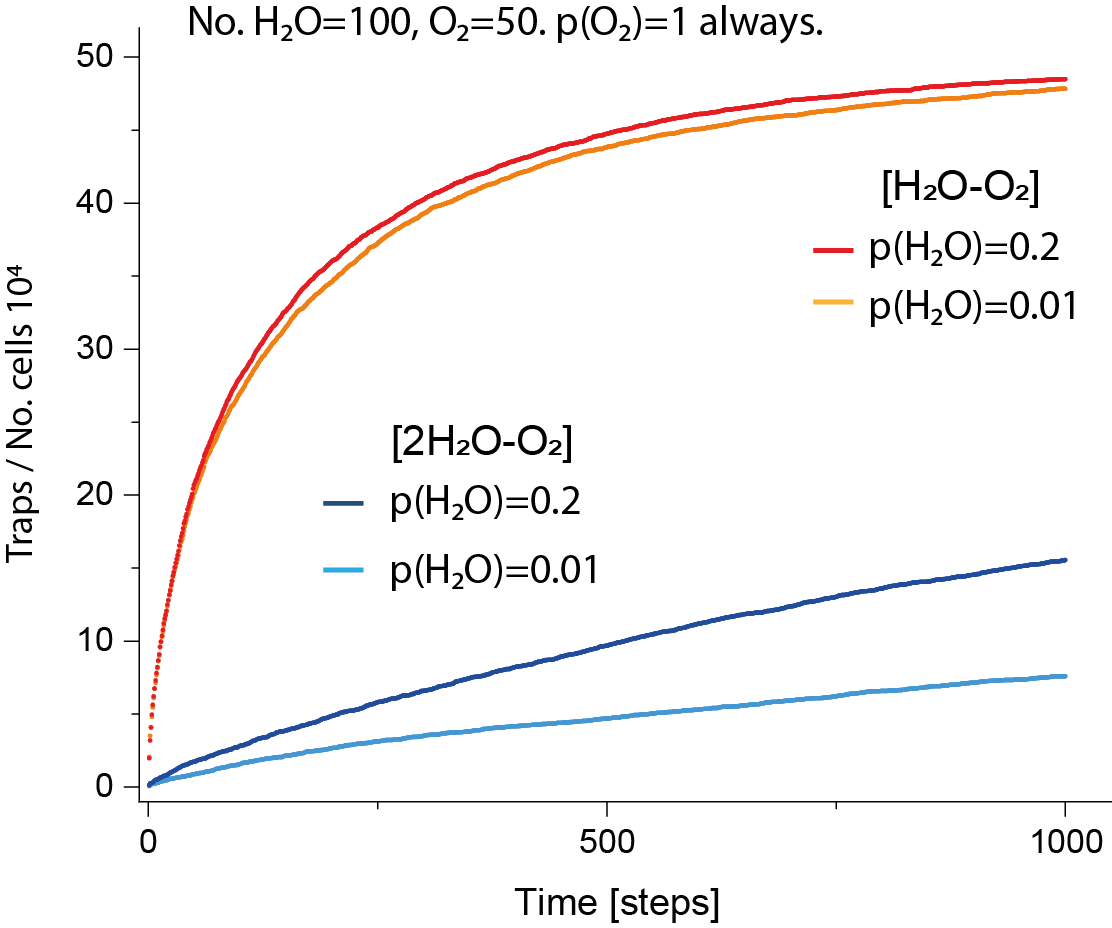


**Figure S10.** Comparison of the influence of the diffusion constants on the trap kinetics. The diffusion constant corresponds to the probability p to make a move to an adjacent cell.

The dynamics of trap formation is largely independent of the diffusion constant.


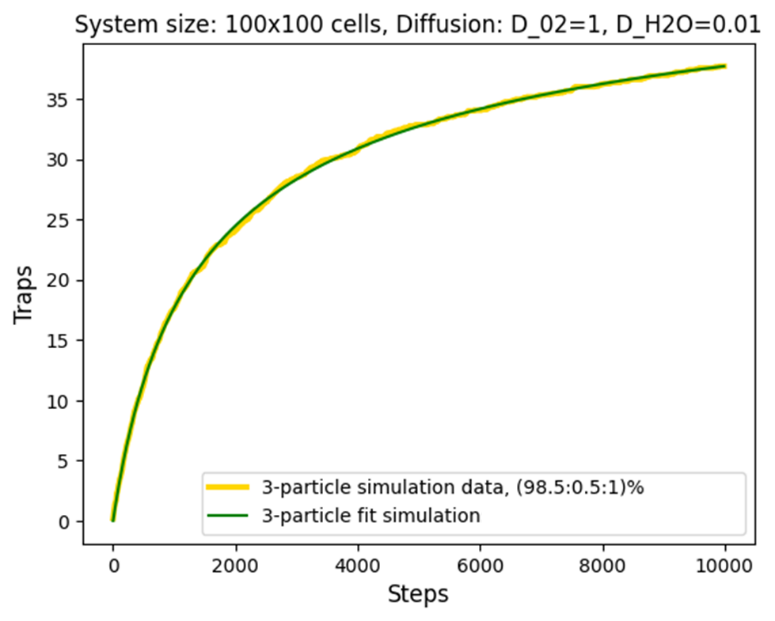


**Figure S11.** The simulated 3-particle trap dynamics was fitted to equation (3) in Scheme 1 of the main text. In the fit, *a* and k were free simulation parameters. In this simulation (R^2^ = 0.9997), the maximum number of traps amounts to *n*(t=∞) = 50, and the fit result *n*_fit_(t=∞) = 51.2 confirms the simulated 3^rd^-order kinetics.

**Fit of trap density to different reaction kinetics**

**
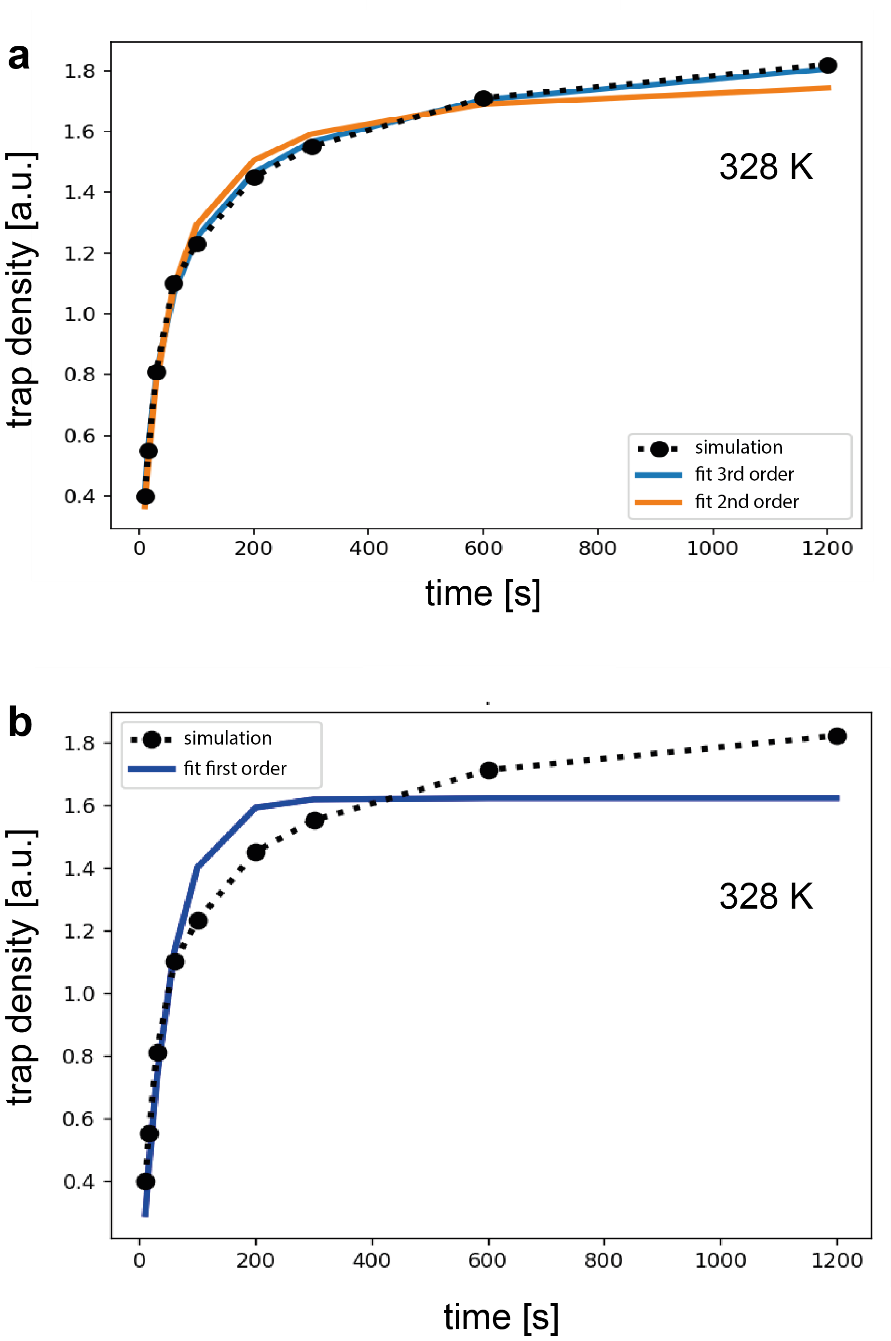
**

**Figure S12**. a) Fits of the simulated trap densities at 328 K over time (Figure 1b, main text) demonstrate that a 3^rd^-order kinetics (R^2^ = 0.999) fits better than a 2^nd^-order kinetics (R^2^ = 0.991). b) the data cannot be fitted assuming a 1^st^-order kinetics.

The 2^nd^-order kinetics was fitted with $n\left( t \right)=\frac{ka_{0}^{2}t}{1+ka_{0}t}$ , the first order kinetics

with $n\left( t \right)=a_{0}\left( 1-ⅇ^{-kt} \right)$.

**Removing water from the sample**


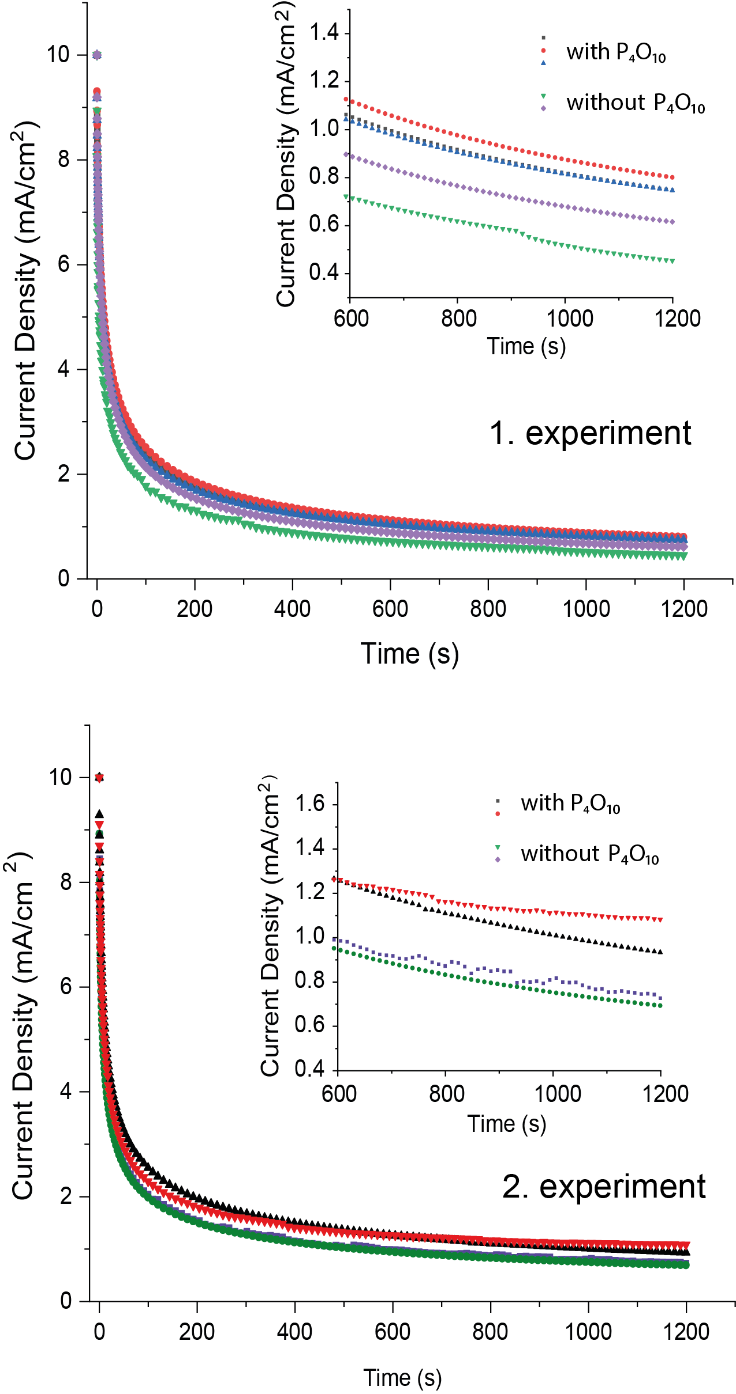


**Figure S13.** We performed experiments to further remove water from electron-only samples. Therefore, a device was stored (in a desiccator) in the glovebox near a powder of P_4_O_10_ for several days, a reference device was stored in the same glovebox away from the desiccant. Measurements show that the current decay of dried samples is indeed smaller than the decay of non-dried samples, visible at longer run-times between 600 s and 1200 s. We observed this trend from two batches of independent samples. The effect is small and we suppose there are two reasons for this. First, for measuring the dried sample, the sample had to be taken out of the desiccator and had to be placed in the atmosphere of the glovebox (O_2_ < 5 ppm, H_2_O < 1 ppm) on the measurement stage. The time between opening the desiccator and terminating the experiment was around 25 minutes, and we suppose that during this time a fraction of H_2_O from the glovebox atmosphere diffused into the dried sample and thereby increased the impurity content again [21].

A second effect to consider is that the influence of a certain number of traps *n* added to the sample strongly depends on the number of traps already present. The influence of the first fractions *n(t)* on the current decay is large, but with increasing number of traps in the device, the influence of further fractions *n(t)* gets small. The current after 20 minutes run-time for the dried samples is reached for the not dried samples after around 500 s. This corresponds for the not dried samples to simulated trap densities of 1.3 x 10^17^ cm^-3^ (after 500 s) and to 1.6 x 10^17^ cm^-3^ (after 20 minutes). This means that by drying the amount of water in the samples, and thereby the trap density, was reduced by around 20%.

**References**

[1] M. Kuik, G.-J. A. H. Wetzelaer, H. T. Nicolai, N. I. Craciun, D. M. De Leeuw, P. W. M. Blom. *Adv. Mater.* **2014**, *26*, 512.

[2] S. Stolz, M. Petzoldt, S. Dück, M. Sendner, U. H. F. Bunz, U. Lemmer, M. Hamburger, G. Hernandez-Sosa. *ACS Appl. Mater. Interfaces* **2016**, *8*, 12959.

[3] M. Diethelm, A. Schiller, M. Kawecki, A. Devižis, B. Blülle, S. Jenatsch, E. Knapp, Q. Grossmann, B. Ruhstaller, F. Nüesch, R. Hany. *Adv. Funct. Mater.* **2020**, *30*, 1906803.

[4] H. T. Nicolai, M. Kuik, G. A. H. Wetzelaer, B. de Boer, C. Campbell, C. Risko, J. L. Brédas, P. W. M. Blom. *Nat. Mater.* **2012**, *11*, 882.

[5] W. F. Pasveer, J. Cottaar, C. Tanase, R. Coehoorn, P. A. Bobbert, P. W. M. Blom, D. M. de Leeuw, M. A. J. Michels. *Phys. Rev. Lett.* **2005**, *94*, 206601.

[6] Q. Niu, G.-J. A. H. Wetzelaer, P. W. M. Blom, N. I. Crăciun. *Adv. Electron. Mater.* **2016**, *2*, 1600103.

[7] M. Diethelm, M. Bauer, W.-H. Hu, C. Vael, S. Jenatsch, P. W. M. Blom, F. Nüesch, R. Hany. *Adv. Funct. Mater.* **2022**, *32*, 2106185.

[8] A. Landi, A. Landi, A. Velardo, A. Peluso. *ACS Appl. Energy Mater.* **2022**, *5*, 10815.

[9] L. Poulsen, I. Zebger, M. Klinger, M. Eldrup, P. Sommer-Larsen, P. R. Ogilby. *Macromol.* **2003**, *36*, 7189.

[10] M. Klinger, L. Poulson Tolbod, K. V. Gothelf, P. R. Ogilby. *ACS Appl. Mater. Interfaces* **2009**, *1*, 661.

[11] L. Poulsen, P. R. Ogilby. *J. Phys. Chem. A* **2000**, *104*, 2573.

[12] S. Shoaee, J. R. Durrant. *J. Mater. Chem. C* **2015**, *3*, 10079.

[13] H. Hintz, H.-J. Egelhaaf, L. Lüer, J. Hauch, H. Peisert, T. Chassé, *Chem. Mater.* **2011**, *23*, 145.

[14] L. Silva, S. Tognana, W. Salgueiro. *Polymer Testing* **2013**, *32*, 158.

[15] E. Linde, N. H. Giron, M. C. Celina. *Polymer* **2018**, *153*, 653.

[16] T. Shigetomi, H. Tsuzumi, K. Toi, T. Ito. *J. Appl. Pol. Sci.* **2000**, *76*, 67.

[17] I. Linossier, F. Gaillard, M. Romand, J. F. Feller. *J. Appl. Pol. Sci.* **1997**, *66*, 2465.

[18] E. M. Davis, Y. A. Elabd. *J. Phys. Chem. B* **2013**, *117*, 10629.

[19] J. P. Goodelle, R. A. Pearson, M. M. Santore. *J. Appl. Pol. Sci.* **2002**, *86*, 2463.

[20] I. Merdas, F. Thominette, J. Verdu. *J. Appl. Pol. Sci.* **2000**, *77*, 1439.

[21] G. Zuo, M. Linares, T. Upreti, M. Kemerink. *Nat. Mater.* **2019**, *18*, 588.
